# Population expansion and genomic adaptation to agricultural environments of the soybean looper, *Chrysodeixis includens*

**DOI:** 10.1111/eva.12966

**Published:** 2020-04-19

**Authors:** Cleane S. Silva, Erick M.G. Cordeiro, Julia B. de Paiva, Patrick M. Dourado, Renato A. Carvalho, Graham Head, Samuel Martinelli, Alberto S. Correa

**Affiliations:** ^1^ Department of Entomology and Acarology Luiz de Queiroz College of Agriculture University of Sao Paulo Piracicaba Brazil; ^2^ Bayer Crop Science Sao Paulo Brazil; ^3^ Bayer Crop Science Chesterfield MO USA

**Keywords:** demography, insect adaptation, landscape genetics, Noctuidae, selection

## Abstract

Evolutionary studies of insect pests improve our ability to anticipate problems in agricultural ecosystems, such as pest outbreaks, control failures, or expansions of the host range. Here, we investigated the mechanisms underlying the evolutionary processes behind the recent census size expansion and local adaptation of *Chrysodeixis includens*. First, we sequenced mitochondrial markers to conduct a phylogeographic investigation of *C. includens* historical processes. Then, we combined a de novo genotyping‐by‐sequencing approach with a study of agricultural landscapes to uncover recent processes of adaptation. Primarily, we found low genetic diversity across all markers and clear indications of a recent demographic expansion. We also found a lack of significant isolation by distance (IBD), and weak or absent genetic structure considering geographic locations. However, we did find initial signs of population differentiation that were associated with host plant types (i.e., soybean and cotton). Agricultural landscape attributes, including soybean crops, were significantly associated with putative markers under positive selection. Moreover, positive selection associated with host differentiation was putatively linked to digestive enzymes. This study showed how landscape composition and host plants can affect the evolutionary process of agricultural pest insects such as *C. includens*.

## 1 INTRODUCTION

Detection of early adaptive changes in insect pest populations is in the best interest of farmers and agroindustry and could allow us to anticipate—rather than merely remediate—agricultural problems such as host range expansions or pesticide resistance. However, there is no clear connection between population dynamics (i.e., minor pests becoming major pests) and the underlying adaptive process, mainly because of differences in the time scales on which ecological and evolutionary dynamics operate (Cavender‐Bares, Kozak, Fine, & Kembel, 2009; Lu et al., 2010). For instance, changes in habitat composition or configuration caused by agricultural practices trigger adaptive responses to the new environments that are not easily noticed in the short term. However, confounding factors do not always stem from landscape changes, and a population's history and dynamics (i.e., recent speciation events, gene flow, genetic drift, and population demography) can also make it difficult to detect early signs of local adaptation.

Agroecosystem configuration and composition may be responsible for sudden changes in ecological and evolutionary dynamics, imposing strong selective pressure and leading to new forms of pests (Altieri, 1999; Alvarado‐Serrano, Van Etten, Chang, & Baucom, 2019). This complex scenario can be accentuated in countries such as Brazil, where agriculture has rapidly intensified and expanded geographically to the Cerrado region (Brazilian savannas) in the past 50 years, compared to places where agriculture is long established (Arvor, Meirelles, Dubreuil, Bégué, & Shimabukuro, 2012; DeFries, Herold, Verchot, Macedo, & Shimabukuro, 2013). However, the cost of this profound economic transformation is the loss and fragmentation of large portions of the native vegetation (Landis, 2017; Ribeiro, Metzger, Martensen, Ponzoni, & Hirota, 2009). The result is extensive uniform cultivation areas where only one or a few crop species dominate the landscape. Beginning in the 1970s, the development of new farming technologies, including new crop varieties and better soil management, allowed farming in new areas (Arvor et al., 2012; Müller, Rufin, Griffiths, Barros Siqueira, & Hostert, 2015). In addition to leading to large areas with only one or a few crops, reducing landscape heterogeneity, the use of crop varieties with early and extra‐early cycles made possible two or three crop seasons annually, increasing the challenges for insect management. The succession of suitable hosts cultivated on such large scales can lead to the rapid evolution of insect pest traits such as expansion of the number of host species, insecticide/*Bt* resistance, and specialization (Andrade et al.., 2016; Santos, Specht, Carneiro, Paula‐Moraes, & Casagrande, 2017). Furthermore, a continuous agriculture landscape may allow increased gene flow among meta‐populations.

In recent decades, the soybean looper, *Chrysodeixis includens* (Walker, [1858]) (Lepidoptera: Noctuidae: Plusiinae), a native to the Americas, has emerged as a serious pest of soybean and cotton (Panizzi, 2013; Santos et al., 2017). This species is polyphagous and shows high fecundity and rapid development (egg‐adult period of 30 days in tropical regions). Little is known about its dispersal abilities. Until recently, *C. includens* was described as a minor pest that rarely reached levels requiring control (Kogan, Turnipseed, Shepard, Oliveira, & Borgo, 1977; Panizzi, 2013). Because of its higher population levels during the last decade, *C. includens* has attracted more attention and become a major concern for growers in many parts of Brazil.

Recent studies using inter‐simple sequence repeat (ISSR) markers in Brazilian populations of *C. includens* have found low genetic diversity and no genetic structure across the area investigated (Palma, Maebe, Guedes, & Smagghe, 2015). However, not much is known about the adaptive potential of the looper in response to the recent expansion of farming to central and northern Brazil. Here, we used sequence fragments of the genes cytochrome c oxidase subunit I (COI) and cytochrome c oxidase subunit II (COII), in addition to genotyping by sequencing (GBS), to investigate first, if populations are locally adapted, and second, the mechanisms underlying the evolutionary processes behind local adaptation in croplands in Brazil. Our explicit goals were (a) to use mitochondrial markers to reveal details of the *C. includens* phylogeographic history in the Americas, (b) to measure the levels of population genetic structure in important agricultural areas in Brazil, and (c) to test whether positive selection is taking place in different geographic regions and cultivated crops; and if confirmed, then to investigate which environmental features are associated with the ongoing adaptive processes. In a broader context, our goal was to shed more light on the adaptive processes that allow previously unimportant insects to become significant pests.

## 2 MATERIALS AND METHODS

### 2.1 Insect sampling and DNA extraction

Specimens of *C. includens* were collected from 15 of the principal common bean, soybean, and cotton‐producing regions in Brazil between January and May 2017, by actively collecting larvae or with pheromone traps. Approximately 800 larvae of *C. includens* were collected per sample site in the field. After they matured, 30 insects were transferred to individual 1.5‐ml Eppendorf tubes containing 99% EtOH and stored at –80°C until further sample processing. Considering the geographic range of soybean and cotton cultivation, we directed our sampling efforts to cover the most ecologically or climatically diverse combination of natural areas (the Atlantic Forest, Brazilian Cerrado, and Caatinga domains) and crop types that could provide a realistic view of the Brazilian agriculture mosaic (Table 1 and Appendix S1).

**TABLE 1:** Information about sampled locations of 12 study areas in Brazil for SNP marker sequencing. Two locations (BACO and MTCV) were sampled during both the soybean‐ and cotton‐growing seasons. *N*_GEN_ refers to the number of insects successfully sequencing using SNP markers

Ecoregion	Location	Code	Latitude	Longitude	Crop	*N*_GEN_
Atlantic Forest	Coxilha, RS	RSCO	28°10'54"S	52°44'46"W	Soybean	16
Atlantic Forest	Pitanga, PR	PRPI	24°17'23"S	52°34'16"W	Soybean	14
Atlantic Forest	Casa Branca, SP	SPCB	21°43'34"S	47°09'09"W	Soybean	13
Cerrado	Araguari, MG	MGAR	18°59'23"S	47°33'52"W	Soybean	15
Cerrado	Mineiros, GO	GOMI	17°37'43"S	52°36'52"W	Soybean	13
Cerrado	Campo Grande, MS	MSCG	20°42'31"S	54°30'03"W	Soybean	14
Cerrado	Campo Verde, MT	MTCVS	15°25'34"S	54°48’05"W	Soybean	10
Cerrado	Campo Verde, MT	MTCVC	15°36'40"S	55°14'05"W	Cotton	10
Caatinga	Correntina, BA	BACOS	11°49'21"S	46°10'52"W	Soybean	10
Caatinga	Correntina, BA	BACOC	13°40'39"S	45°41'21"W	Cotton	10
Caatinga	Russas, CE	CERU	04°55'28"S	38°00'14"W	Bean	13
Caatinga	Teresina, PI	PITE	05°02'21"S	42°47'22"W	Bean	12

DNA was extracted from the insects by using an adapted CTAB (2%) extraction protocol and resuspended in 70 µl of Milli‐Q water (Doyle & Doyle, 1987). DNA extraction quality control was first performed by visual inspection in agarose gel (0.8% w/v), followed by spectrophotometric and fluorometric methods. Samples were tested in a NanoDrop spectrophotometer and agarose gel to check for contaminants and DNA integrity, respectively, followed by a Qubit assay to estimate the DNA concentration in each sample. Only samples with a concentration above 15 ng/µl were included in the subsequent steps.

### 2.2 Landscape and landscape genetic analyses

We used only satellite images with a maximum of two months’ difference between the sampling time and the satellite image capture. This criterion avoided problems with cloudy or unavailable images, thus improving the landscape classifications. We used images from two satellites, CBERS 4 and LANDSAT 8, available from The National Institute of Spatial Research (INPE) and the United States Geological Survey EarthExplorer (USGS EarthExplorer) repositories, respectively. A buffer of a 25‐km radius from the sampled point was used to delimit the size of the classified landscapes used. Landscapes were classified in the software ArcGIS 10.2.2, using the “create signature” option that uses different spectral responses that can be associated with the presence of a class (i.e., agriculture, native vegetation, urban areas, and exposed soil). Supervised classification was performed using a maximum‐likelihood classification and was manually inspected for errors and misclassifications. At two geographic locations (BACO and MTCV), two sample collections were performed (soybean versus predominantly cotton areas) in overlapping landscapes. We only performed the analyses on the soybean landscape at those two locations, to ensure a minimum difference in the time frame between sample collections, avoiding the effect of migration between the landscapes. Soybean and cotton landscapes were contrasted later in separate analyses.

We used the LecoS plug‐in, which is based on metrics taken from FRAGSTATS v4, to estimate the landscape metrics based on the composition of each attribute in the landscape (Jung, 2016; McGarigal, Cushman, Neel, & Ene, 2012). For “patch‐based metrics” (e.g., bare soil, native forest, soybean), land cover, landscape proportion, edge length, edge density [ED = (Total length (m) of the edge in landscape/ Total landscape areas m^2^) *10,000], number of patches, and mean patch area were collected to describe landscape structure and composition. For the “landscape‐level metrics,” the Shannon diversity index (SHDI) and Shannon evenness index (SHEI) were estimated (Shannon & Weaver, 1949).

The SHDI is a measure of diversity that is commonly applied in community‐ecology studies but can also be used to measure diversity in landscapes. When the SHDI is zero, the landscape contains only one patch, and therefore, no diversity is observed at that particular scale. As the value of the SHDI increases, a larger number of different patch types are observed, and consequently, a higher patch richness. A second index used here was the SHEI, which expresses the degree of dominance of a patch type in relation to other patch types present in the landscape. Low values can be interpreted as meaning that the landscape is dominated by one or a few patch types, whereas high values mean that patch types are evenly distributed in the landscape.

Principal component analysis (PCA) was used to reduce the dimensionality and to simplify pattern recognition of the landscape and climate variables. We selected land cover as our patch‐based metric to study the response to the landscape composition at the genomic level. The 14 land‐cover attributes were transformed with PCA to reduce the dimensionality, and the first PCA coordinate was used as a predictor. We selected land cover as our primary landscape variable based on our a priori knowledge that *C. includens* strongly responds to host availability in the field (Andrade et al., 2016; Santos et al., 2017). The same approach was also used for the climate variables; 19 WorldClim variables (www.worldclim.org/bioclim) had their dimensional space reduced by PCA transformation. This approach allowed us to assess the position of each putative population in environmental space (climate + hosts). We used the coordinates of the projections on the first PC axis as our independent variables. The importance of each variable in the PCA was presented as the variable contribution to the first PC axis, which was given by squaring the product of the loadings by the component standard deviation and dividing it by the square of the total PCA loadings multiplied by the component standard deviation (Kassambara, 2017).

### 2.3 Mitochondrial COI and COII amplification and sequencing

Two mitochondrial fragments from the COI and COII genes were amplified in PCR for 61 individuals from 15 sample sites (Appendix S1) (Monteiro et al., 1999; Simon et al., 1994). Raw mitochondrial COI and COII sequence chromatograms were inspected and edited to 522 bp (NCBI accession: MK256487 – MK256546) and 697 bp (NCBI accession: MK256547 – MK256606), respectively, using Sequencher 4.0.1 (Gene Codes Corp.). Next, the COI and COII sequences were concatenated and aligned using MUSCLE with the default options in MEGA 7.0 (Kumar, Stecher, & Tamura, 2016).

### 2.4 GBS library preparation and sequencing

Two libraries containing 75 samples each (150 insects in total), from 12 sample sites (Table 1), were processed using the GBS protocol (Elshire et al., 2011). Samples from three locations did not meet the quality standards and were therefore not included in the GBS library preparation. Only one enzyme (*Pst*I, CTGCAG) was used during the genome digestion step to reduce genome complexity. After library preparation, DNA samples were quantified by qPCR, and the size distribution checked using an Agilent BioAnalyzer. Libraries were sequenced on an Illumina HiSeq 2500 (100‐bp single‐end reads) at the Animal Genome Centre at USP/ESALQ (see Appendix S3 for details).

### 2.5 mtDNA sequence analysis

For COI‐COII concatenated sequences, we calculated the haplotype diversity and nucleotide diversity parameters using DnaSP ver. 5 (Librado & Rozas, 2009). The analysis of molecular variance (AMOVA), using the samples collected as a putative population, was also carried out using Arlequin with parametric bootstrapping (1,000 replicates) and significance at the 5% level (Excoffier, Smouse, & Quattro, 1992). A network of median‐joining haplotypes was generated using the PopART software program to reconstruct the genealogical relationships among the COI‐COII haplotypes. The hypothesis of demographic expansion was tested using Tajima's D and Fu's Fs tests of selective neutrality in Arlequin 3.1 (Excoffier, Laval, & Schneider, 2006). Both tests used 1,000 random samples, using coalescent simulations and the “Infer from distance matrix” option for “Haplotype definition.” We also tested the goodness of fit of the observed mismatch distribution to that expected under the spatial expansion model. The sum of squared deviations (SSD) and Harpending's Raggedness index statistics and their associated p‐values were calculated using Arlequin 3.1 (Excoffier et al., 2006) with 1,000 replicates. Finally, we used additional COI sequences downloaded from BOLD Systems to estimate *C. includens* COI haplotype relationship and genetic diversity throughout the Americas. Details of the method are shown in Appendix S2.

### 2.6 GBS data analysis

Raw sequences were demultiplexed, trimmed to 90 bp, and filtered according to the *Phred* scores using *process‐radtags* in stacks 2.2, using default settings (Catchen, Hohenlohe, Bassham, Amores, & Cresko, 2013). We proceeded with the analysis using the five major modules available in STACKS in a de novo approach. During the first step, the reads were used to build RAD loci (restriction site‐associated DNA marker loci) using *ustacks* to form putative loci present in each sample file. At this stage, two critical parameters are the minimum number of raw reads to form a putative allele (*m* = 3) and the number of mismatches allowed between stacks to merge them into a single allele (*M* = 4). Setting m to 3 has shown good performance for a broad range of data and represents a good trade‐off between coverage and polymorphs (Paris, Stevens, & Catchen, 2017; Rochette & Catchen, 2017). The criterion to merge stacks into a single locus (M) is highly dependent on the data and required a balance on the homolog and paralogue loci detection. In general, the presence of a higher degree of polymorphs requires higher values for M, but here we tested a range of values (M1–M8) to identify where the parameter plateaus (Paris et al., 2017), which proved to be ~4 (Figure S3). Next, STACKS creates a catalog containing consensus loci using *cstacks*, using 4 as the number of mismatches allowed between sample loci (*n* = 4). We used the general criteria of *M* = *n*. In the following step, STACKS matches loci found in each individual against the catalog, using default settings. Using this approach, STACKS genotyped a total of 340,089 loci with mean effective per‐sample coverage of 97.7× (stdev = 27.9×), minimum coverage 10.1×, and maximum coverage 164.8×.

Last, we used the *population* module to filter missing data, retaining only loci present in 80% or more individuals within a sampling location (*r* = .8) and ~80% or more across sampling locations (*p* = 8). Minor allele frequency cutoff was set at 0.05, and maximum observed heterozygosity limited to 50%. The resulting dataset was saved as VCF, genepop, and structure files and converted to other formats using PGDSPIDER 2.0 when necessary (Lischer & Excoffier, 2012). The percentage of missing data allowed and the number of markers implemented to estimate parameters can significantly affect estimates of genetic diversity; therefore, we calculated genetic diversity parameters varying both the overall percentage of missing genotypes and the number of SNPs to find the most stable values. In our dataset, the most stable estimates (i.e., that stop varying as we increase the number of SNPs used) were acquired with more than 5,000 SNPs, while no more than 80% of overall missing genotypes were allowed (see Appendix S3).

### 2.7 Demographic history

We reconstructed the demographic history of *C. includens* using the multi‐locus method Extended Bayesian Skyline Plot (EBSP) implemented in BEAST version 1.8.2 (Bouckaert et al., 2014; Drummond, Suchard, Xie, & Rambaut, 2012). Because of the lack of genetic structure found in the *F*_ST_ and structure analysis, we randomly selected 50 neutral sequences (not flagged in our analysis as putative candidates under selection) from a simple pooled population to perform the demographic reconstruction analysis (Calderón et al., 2016). Site model and clock model partitions were linked, whereas the tree partitions remained unlinked. The HKY substitution model was implemented because it allowed us to work with fewer parameters. A strict clock model was implemented using a fixed mutation rate across the genome of 2.9 × 10^−9^ mutations/site/generation. The mutation rate was selected based on similar organisms, as no value is available for this species (El‐Shehawi & Elseehy, 2016; Keightley, Ness, Halligan, & Haddrill, 2014; Oppold & Pfenninger, 2017). We assumed that the species has 10 generations per year. The chain length of 100 million generations was sampled every 4,000 states, and a 20% burn‐in was implemented in three independent runs. The results were inspected in Tracer, examining the posterior ESS (effective sample size) and the convergence among the runs.

### 2.8 *F*_ST_, population structure, and gene flow

We assessed genetic partitioning by means of the software STRUCTURE, using a single SNP per locus (i.e., ‐‐write_single_snp was used in population module in STACKS) (Pritchard, Stephens, & Donnelly, 2000). An initial burn‐in of 1.5 × 10^5^ was used, followed by 5 × 10^5^ Markov chain Monte Carlo (MCMC) interactions, using 10 replications for each K value without prior population information. The analyses were run from *K* = 1 to *K* = 10, allowing allele correlation and an admixture model. The most likely number of clusters was determined using the Evanno method implemented in STRUCTURE HARVESTER 0.6.93 (Earl & vonHoldt, 2012). The STRUCTURE results were averaged using CLUMPP (Jakobsson & Rosenberg, 2007) and visualized using DISTRUCT (Rosenberg, 2004). Additionally, we used principal component analysis (PCA) implemented in *ade4* and *adegenet* to investigate the underlying structure.

Pairwise differentiation between sampling locations was estimated based on *F*_ST_ estimation calculated in STACKS (Weir, 1996). A heatmap was produced to illustrate the *F*_ST_ relationships, using the R program for both the complete set of SNPs and outliers. To test the correlation between genetic distance and geographic distance, a Mantel test, including an all‐pairwise‐comparison matrix, was performed with 10,000 permutations, using the R libraries *ecodist* (Goslee & Urban, 2007) and *ade4* (Dray & Dufour, 2007).

### 2.9 Outlier detection and associating genomic variation with environmental characteristics

We used Lositan to detect putative loci under natural selection. The method implemented in Lositan simulates the joint distribution between *F*_ST_ and heterozygosity (*H*_e_) under neutrality (Antao, Lopes, Lopes, Beja‐Pereira, & Luikart, 2008), assuming the island model of migration with neutral markers. The neutral mean *F*_ST_ was calculated using 100,000 simulations, with a confidence interval of 0.99 and a false discovery rate (FDR) of 0.05. To assure more stringent detection parameters, we selected the “force mean *F*_ST_” and “neutral mean *F*_ST_” options. We also used a Bayesian approach that estimated the posterior probability of a given locus to be under selection, contrasting this assumption with the neutral model. A second analysis was implemented in BayeScan v2.1 (Foll & Gaggiotti, 2008). A total of 20 pilot runs with 5,000 interactions each were generated before computing Markov chains. Burn‐in was set to 50,000 steps, and the chains had 5,000 interactions with a thinning interval of 10. We considered prior odds of 10 and a threshold of a posterior P of >0.95 to select an SNP as “under positive selection.” In both analyses, we contrasted sampling locations, except the cotton areas (BACOC and MTCVC) that partially overlapped in time and space with soybean areas (BACOS and MTCVS). Contrasts between soybean locations and cotton locations were performed separately.

We used a series of partial Mantel tests with 10,000 randomizations to test the correlation between the variation in *F*_ST_ outliers (putative loci under directional selection) detected by both Lositan and BayeScan, and landscape and climate variables. The partial Mantel test accounts for the nonindependence of points by creating a random distribution to use as a null distribution and test for significance (Mantel, 1967). Additionally, a partial correlation for each variable is calculated by controlling for any variables already in the model. Four hypotheses may explain the driver(s) of outlier RAD locus divergence: (a) isolation by habitat hosts and habitat composition (landscape distance), (b) isolation by the environment (IBE) due to climate differences, and (c) isolation by geographic distance (IBD).

Additionally, the presence of loci that correlate with environmental variables (climate and landscape composition) was also tested using a latent factor mixed‐effect model (LFMM) implemented in R package *LEA* (Frichot & François, 2015). The advantage of the LFMM algorithm is to correlate markers and environmental values while taking into account possible levels of population structure (i.e., latent factors). We defined latent factor 2 (*K* = 2) based on “*snmf*.” Again, for this analysis, individuals collected from cotton landscapes were removed, as were individuals with more than 10% missing data. The two variables were used to test the associations with climate and landscape. To test the association, we used the coordinates of the first PC of WorldClim variables and the landscape composition (percentage of each component of the landscape) described in the previous section (“Landscape and landscape genetic analyses”). The Gibbs sampler algorithm was run for 10,000 cycles, following a burn‐in of 5,000 cycles. To control for false‐positive detection, we used the Benjamin–Hochberg algorithm, adjusting the false discovery rate (FDR) *q* = 10%. Putative loci under selection detected by more than one method were used as queries in a BLASTx search against the NCBI database, using Blast2GO (Götz et al., 2008). Ontologies with *e*‐value <1×10^−3^ were used for annotation.

## 3 RESULTS

### 3.1 Mitochondrial COI and COII variation

**FIGURE 1:**
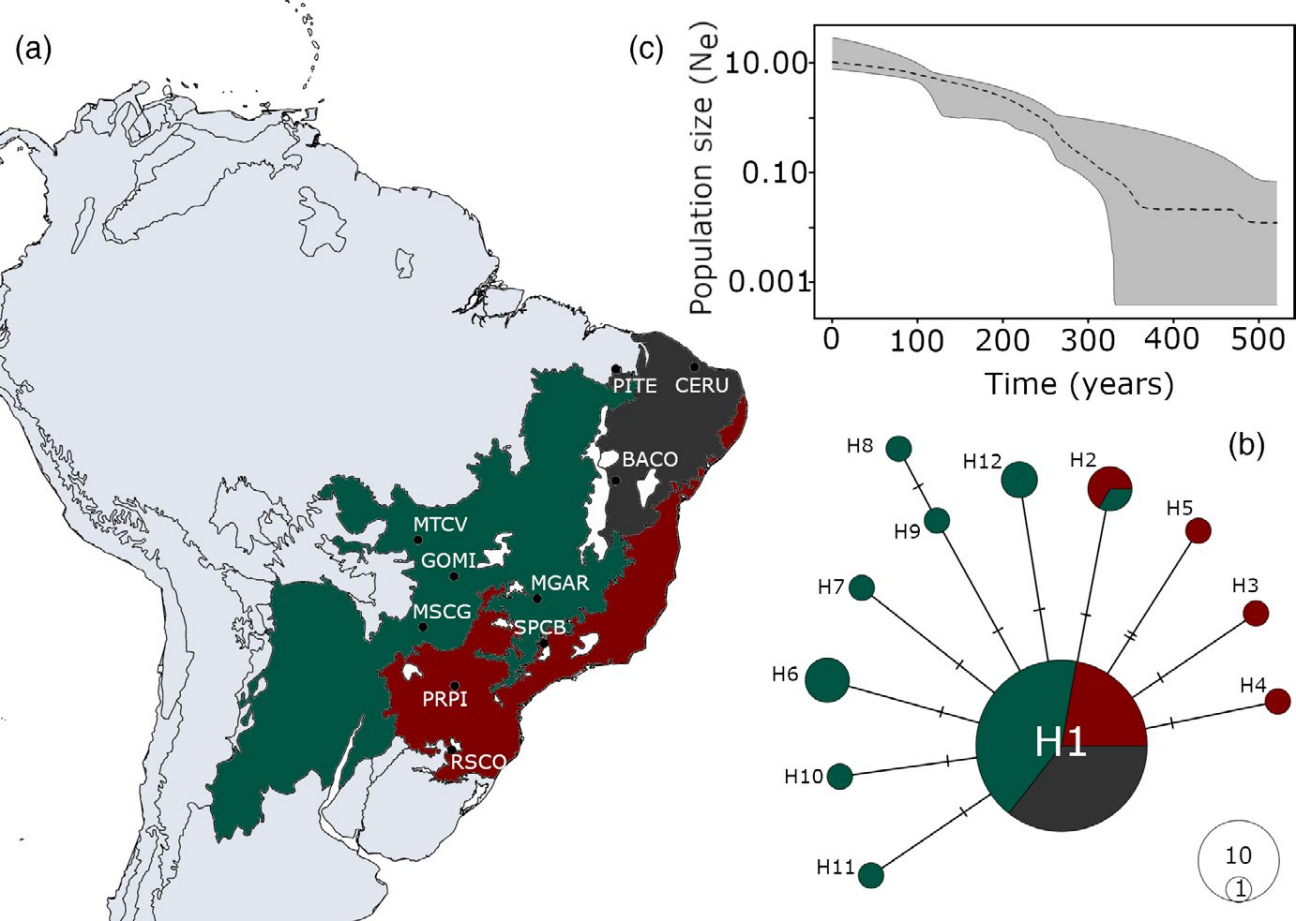
*Chrysodeixis includens* population demography. (a) Geographic distribution of the 12 collection sites of *C. includens* (b) Median‐joining network of *C. includens* haplotypes in Brazil, based on two mitochondrial gene fragments (COI‐COII). The size of each haplotype circle reflects the haplotype frequency, and hatch marks reflect mutation steps. Colors indicate the three Brazilian biomes: Caatinga (Ca), Cerrado (Ce), and Atlantic Forest (AF). (c) Bayesian skyline plot (BSP) showing population size dynamics for *C. includens* in Brazil based on 50 loci randomly sampled throughout the genome. The y‐axis indicates an effective population size (Ne) scaled by mutation rate (μ) as a function of time (years). Black dotted line shows the median BSP estimate, and gray area shows the upper and lower 95% highest posterior density limits. The soybean looper can produce up to 12 generations per year in a tropical climate.

The mitochondrial network (COI‐COII) of Brazilian *C. includens* revealed a star‐like topology of the haplotype relationships, with a central haplotype (haplotype H1) that is widely distributed and in a high frequency, and additional haplotypes at low frequencies (11 total), connected to the central haplotype by a single mutation step (Figure 1b). The ubiquitous haplotype H1 was recovered from 73.8% of the individuals and was present at all sample sites. Eight haplotypes were singletons (i.e., they occurred only once in our dataset), and three were present in low frequency (H2, H6, and H12) and geographically restricted to certain sample sites.

The genetic diversity of *C. includens* in Brazil seems to be evenly distributed through the species’ range and is moderate to low. The haplotype and nucleotide diversities across the locations sampled were *H*_d_ = 0.455 and π = 0.00048, respectively. Haplotype and nucleotide diversities were highest in the Cerrado (*H*_d_ = 0.622; π = 0.00067) and Atlantic Forest biomes (*H*_d_ = 0.505 and π = 0.00059) and lowest in the Caatinga biome, where each sample site had only a single haplotype (Appendix S1). The AMOVA confirmed the lack of genetic structure at the regional spatial scale, that is, the differences within populations accounted for almost all the genetic variance for *C. includens* in Brazil (98.1%; Φ_ST_ = 0.019) (Appendix S4).

Similar trends were observed at a broader scale when 173 COI sequences from 8 different countries were analyzed (Appendix S2). The analysis using curated sequences from the BOLD database also revealed the widespread occurrence of the single dominant haplotype A1, found throughout the Americas. This analysis also showed haplotypes with a lower frequency derived from A1 by a single mutational step (i.e., star‐like topology), similar to the findings in Brazil.

### 3.2 Demographic history

The analyses of both mitochondrial sequences and SNP markers indicated a demographic expansion of *C. includens* in Brazil. Neutrality tests based on COI‐COII indicated a population expansion, with Tajima's *D* (*D* = −2.21; *p* < .001) and Fu's *F*s (*F*s = −12.16; *p* < .001). In addition, the model of a sudden expansion did not reject the hypothesis of a spatial expansion for *C. includens* (SSD = 0.0002; *p* = .60; Raggedness = 0.1090, *p* = .60). Similarly, the EBSP analysis based on 50 nuclear sequences showed a signature of recent demographic expansion in the *C. includens* populations. Based on the assumed molecular clock calibration (2.9 × 10^−9^ mutations/site/generation), the expansion started ~300 years before the present (Figure 1c).

### 3.3 Landscape/environmental composition

**FIGURE 2:**
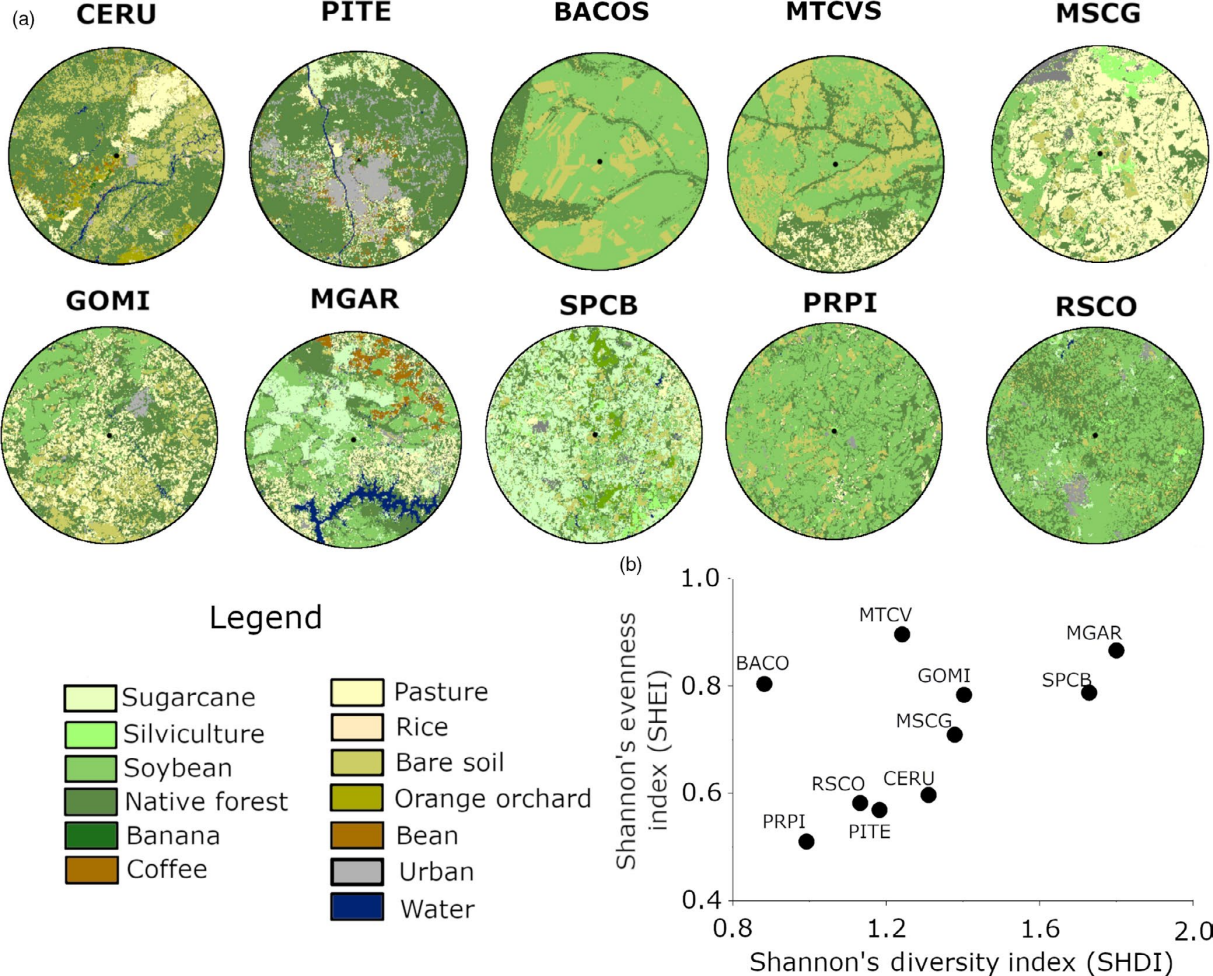
Agriculture landscapes and crop diversity. (a) Landscape classification (25‐km radius buffer) of 10 sampling sites for *Chrysodeixis includens* (in soybean crops) in Brazil. Images were obtained from two satellites, CBERS 4 and LANDSTAT 8, available at the National Institute of Space Research (INPE) and the United States Geological Survey EarthExplorer (USGS EarthExplorer) repositories, respectively. For details, see Appendix S12. (b) Plot of Shannon diversity index (SHDI) against Shannon evenness index (SHEI) for 10 agricultural landscapes where *Chrysodeixis includens* is commonly found in soybean crops in Brazil

There was a tendency for agricultural landscapes with higher Shannon diversity indexes to also show more evenly distributed proportions of vegetation cover types across the different classes, although this relationship was not statistically significant (*r* = .47, *p* = .17) (Figure 2b). Landscapes with a higher diversity of patch‐fragment classes and more evenly distributed patches were found in MGAR and SPCB. The least diverse and least evenly distributed compositions of landscape attributes were found in BACOS (i.e., least diverse) and PRPI (i.e., least even), respectively (Figure 2). Soybean farms were the predominant agriculture class in 80% of the landscapes, ranging from 7% (SPCB) to 52% (PRPI), with a mean proportion of 28.80% across all landscapes (Appendix S5). The second most common class was native forest, ranging from 12% (BACOS) to 51% (PITE), with a mean proportion of 25.75%. Bare soil and native forest showed the most patches, indicating high levels of fragmentation of the native vegetation (Appendix S5). Based solely on the lack of heterogeneity, we can predict that insects from BACO in the state of Bahia and PRPI in the state of Paraná might be under greater host selection pressure due to monoculture, if we consider only land cover and patch size.

**FIGURE 3:**
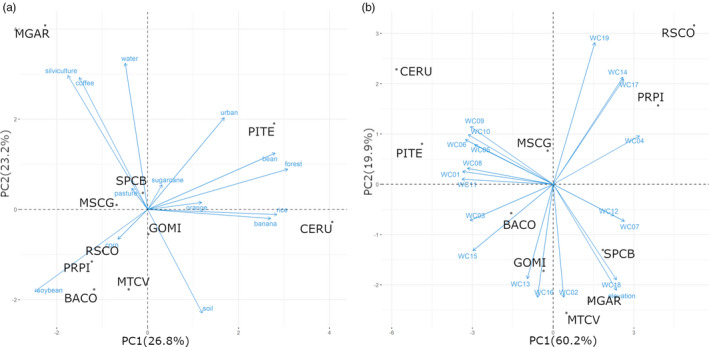
Environmental distance according to landscape composition and climate. Principal component analysis (PCA) loading plots for 15 landscape attributes and 19 WorldClim environmental variables. Solid lines (blue) show the loading of landscape attributes (a) or climate variables (b), and points show the scores for individual landscapes from each site

The PCA of the agricultural landscapes, based on the land‐cover attributes, revealed similarities and dissimilarities between the landscapes and was highly useful for pattern recognition (Figure 3a). The first two components were statistically significant and together explained 50.0% of the overall variance (Appendix S6). The most important factors in the first component (PC1) were native forest (18.65% contribution), rice (15.88%), common bean (15.45%), bananas (14.41%), and soybeans (12.12%). The most important factors in the second PCA component (PC2) were water (23.90%), silviculture (20.03%), and coffee (19.44%). Soybeans were more associated with corn (maize), while coffee was more associated with silviculture and pasture. PRPI and RSCO had similar landscapes, where soybean was the predominant component. MGAR had a unique landscape, where silviculture and coffee were the predominant components.

The PCA of the WorldClim data defined a climate space of reduced dimensionality where the agricultural landscapes are located. The first two components were significant and together explained 80.1% of the overall variance (Figure 3b, Appendix S6). The first component was more associated with temperature (WC01 = “Annual Mean Temperature” and WC11 = “Mean Temperature of Coldest Quarter”), while the second component was more associated with precipitation (WC19 = “Precipitation of Coldest Quarter”). In the climate space, the pairs PRPI and RSCO, MGAR and SPCB, and CERU and PITE occupied very similar environments.

### 3.4 Population diversity and structure

**FIGURE 4:**
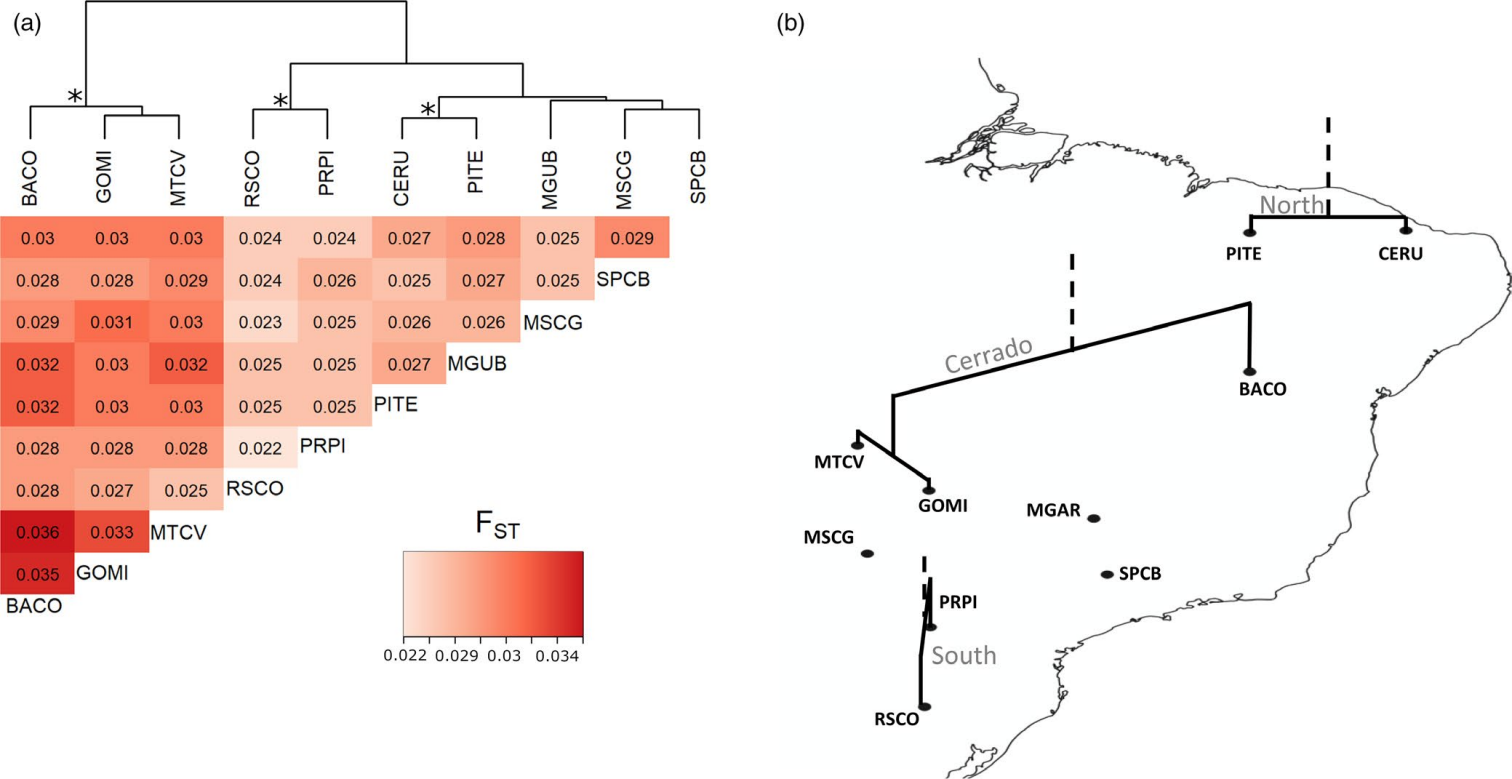
Genetic differentiation according to the fixation index (*F*_ST_) of *Chrysodeixis includens* populations. (a) Heatmap of pairwise *F*_ST_ values based on 1,520 neutral makers. (b) Map showing grouping pattern of samples collected in the Cerrado, North, and South regions according to *F*_ST_ values. *Indicates on the map, clusters according to broad geographic regions (Cerrado, North, and South)

SNP‐estimated diversity indices were largely concordant with the low diversity found in the mitochondrial data, and a narrow range of values for nucleotide diversity (π = 0.006–0.007), observed heterozygosity (*H*_O_ = 0.005 ± 0.000), and inbreeding coefficient (F_IS_ = 0.004–0.007) were found across the 12 sites where samples collected in the same regions were more similar (Table 2). Analysis based on pairwise *F*_ST_ revealed weak structuring (i.e., range of 0.022–0.036 and mean of 0.028), but a consistent separation of collecting sites according to broad regions, where samples collected in the same regions were more similar according to *F*_ST_ values (Figure 4a). However, nonsignificant isolation by distance at the 5% level was detected (*r* = .30, *p* = .06). The STRUCTURE analysis confirmed a low degree of differentiation among the sampling locations. However, a higher value of *K* (*K* = 4) was detected by the Evanno method (Earl & vonHoldt, 2012), even though populations were fully admixed (Appendix S7).

**TABLE 2:** Summary of genetic statistics considering only variant positions in populations of *Chrysodeixis includens* assessed using SNP markers: observed heterozygosity (*H*_o_), expected heterozygosity (*H*_E_), Wright's inbreeding coefficient (*F*_IS_), and nucleotide diversity (π)

Sample location	*H*_0_	*H*_E_	*F*_IS_	Π
RSCO	0.174	0.240	0.247	0.248
PRCM	0.167	0.236	0.243	0.246
SPCB	0.171	0.238	0.239	0.249
MGAR	0.172	0.244	0.251	0.253
GOMI	0.181	0.239	0.222	0.249
MSCG	0.182	0.245	0.240	0.254
MTCVC	0.177	0.218	0.162	0.234
MTCVS	0.170	0.228	0.211	0.241
BACOC	0.171	0.219	0.182	0.236
BACOS	0.175	0.228	0.194	0.244
PITEB	0.178	0.237	0.218	0.247
CERU	0.185	0.245	0.217	0.256

### 3.5 Landscape/Environmental correlation and association study

**FIGURE 5:**
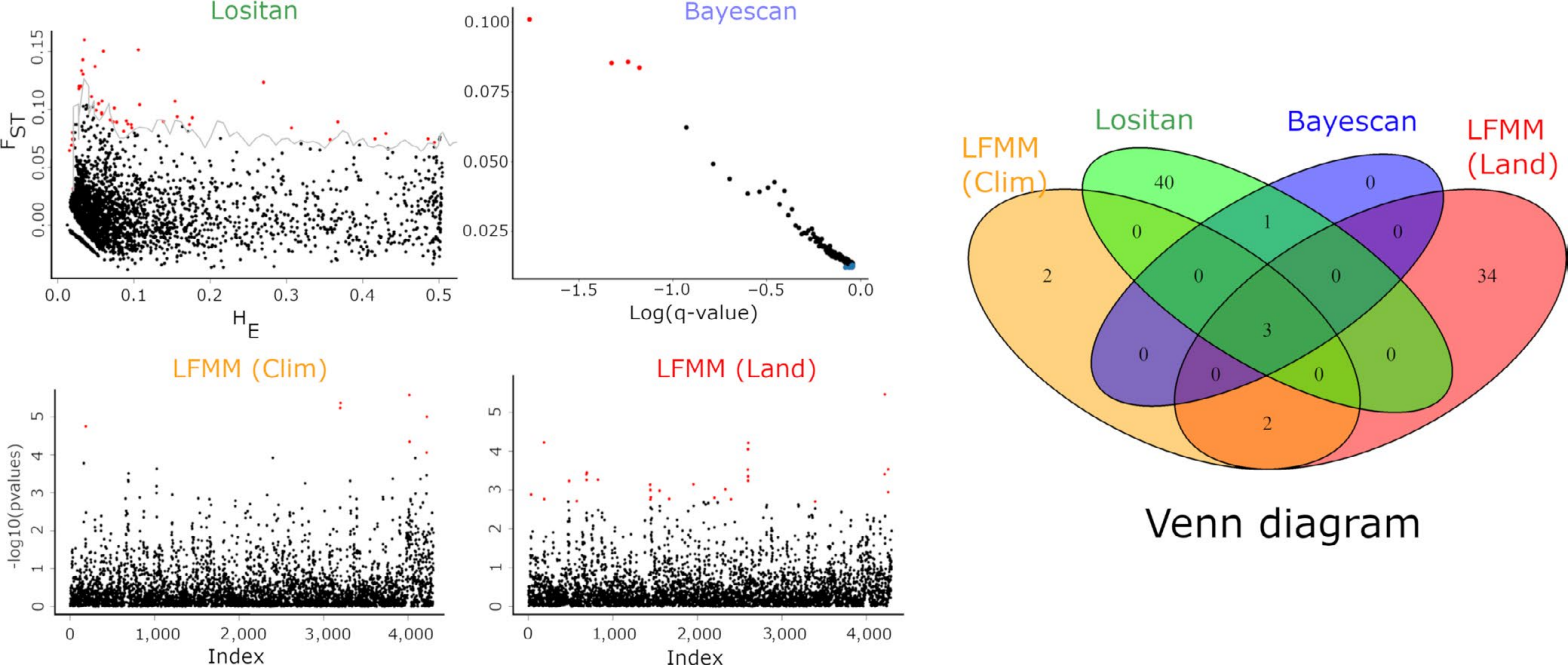
Detection of outlier SNP under positive selection of *Chrysodeixis includens* populations, using three different approaches. Plots showing outlier loci putatively under positive selection (red) detected by (a) Lositan (*N* = 43) and (b) BayeScan (7). Manhattan plot of candidate loci that showed a significant association (red) with (c) landscape variables (*N* = 39) and (d) climate variables (*N* = 7) by the LMFF method. On the right‐hand side, the Venn diagram shows the SNP candidate overlap between the different methods

Lositan yielded 44 loci that are putatively under positive selection, out of the 3,503 SNP evaluated; while BayeScan detected 4 candidates, all present in the Lositan list of candidates (Figure 5). The four shared loci were not identified in a Blastx search. The Mantel tests demonstrated that landscape distance alone correlated significantly with genetic distance, based on *F*_ST_ outliers (putative loci under positive selection) among population pairs, independently of the method used (Table 3). Conversely, environmental distances based on climate variables showed no correlation with the genetic distance, while a significant correlation with geographic distance was present only in Lositan markers. When partial Mantel tests were used to control for secondary factors, a slight increase in the correlation between the landscape distance and genetic distance was observed (Table 3).

**TABLE 3:** Partial Mantel tests for partitioning of genetic variation of 43 markers under directional selection selected by Lositan and 4 markers selected by BayeScan in *Chrysodeixis includens* based on landscape variables, environmental variables, and geographic distance

Models	*r*	*p*‐values
Lositan		
Landscape distance	0.31	.05^a^
Environmental distance	0.19	.12
Geographic distance	0.33	.03^a^
Landscape distance, controlling for environmental distance	0.35	.03^a^
Landscape distance, controlling for geographic distance	0.36	.02^a^
Environmental distance, controlling for landscape distance	−0.06	.64
Environmental distance, controlling for geographic distance	0.04	.32
BayeScan		
Landscape distance	0.47	.01^b^
Environmental distance	0.17	.13
Geographic distance	0.20	.10
Landscape distance, controlling for environmental distance	0.47	.01^b^
Landscape distance, controlling for geographic distance	0.49	.00^b^
Environmental distance, controlling for landscape distance	−0.13	.85
Environmental distance, controlling for geographic distance	0.03	.32

^a^ Significance at 5% probability.

^b^ Significance at 1% probability.

Using a latent factor of 2 and controlling FDR at 10%, we obtained 39 candidate SNPs associated with the landscape variables, and 7 associated with climate variables, using the LFMM approach (Figure 5). The *p*‐values of the putative candidates ranged from .002 to 3.4 × 10^–6^, showing overlap with the Lositan and BayeScan candidates (Figure 5, Venn diagram). From the list of candidates associated with landscapes, 13 were successfully identified in a Blast search, 10 mapped, and 7 annotated. Among potential candidates, a locus associated with glucuronosyltransferase activity was recognized (UDP‐glycosyltransferase). Associated with climate variables, no loci containing outlier SNP were identified.

### 3.6 Soybean versus cotton

**FIGURE 6:**
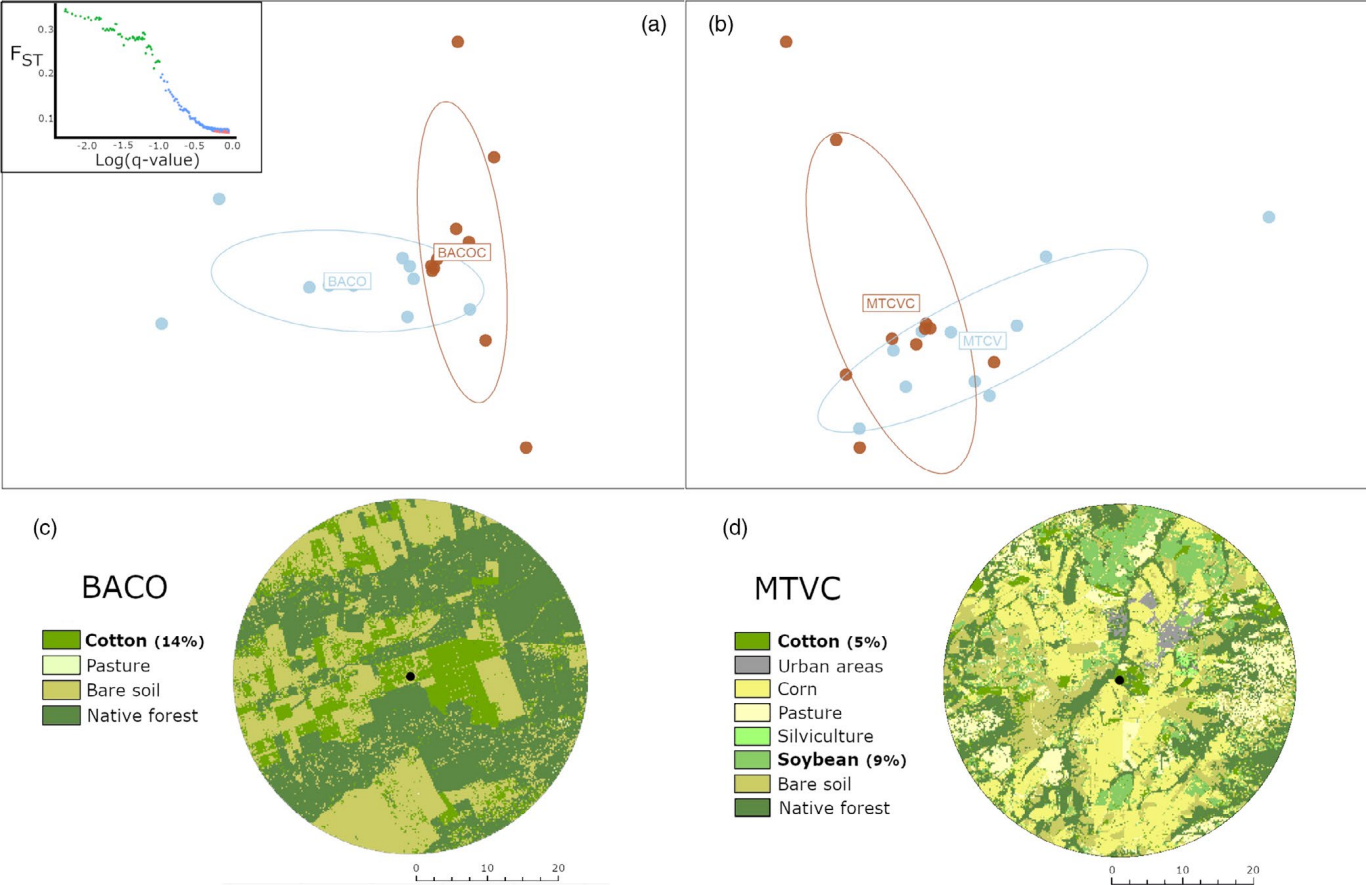
Population differentiation by host plants (soybean versus cotton). (a) Principal component analysis (PCA) using 7,923 SNP markers of *Chrysodeixis includens* collected from soybean and cotton plants in successive growing seasons at BACO, and (b) using 10,010 SNP markers for insects collected at MTCV. The small panel (upper left) shows putative markers under positive selection when cotton versus soybean insects from BACO were contrasted. No sign of selection was found at MTCV. Ellipse colors according to host plants: cotton (brown) and soybean (blue). Figures (c) and (d) show the landscape composition of BACO and MTCV during the cotton season. Samples labeled according to the host type are inside their 95% inertia ellipses

Principal component analysis revealed that larvae collected in cotton fields were slightly different from those collected in soybean fields in BACO (~14% of cotton, distributed in 925 patches), but not in MTCV (~5% of cotton, distributed in 1,259 patches) (Figure 6). When contrasting locations where both cotton and soybean were sampled (i.e., BACO and MTCV), BayeScan found signs of natural selection only in BACO. A total of 43 candidate loci under positive selection were identified by the BayeScan method (Figure 6a, upper‐left corner), of which 19 were successfully identified by Blastx, 4 mapped, and 2 annotated. One of the loci successfully annotated is associated with proteolysis (serine‐type D‐Ala‐D‐Ala carboxypeptidase activity). Other nonannotated Blastx results included aminotransferase, amidases, and carboxypeptidase.

## 4 DISCUSSION

Our results based on the mtDNA and SNP markers provided strong support for the assumption that the *C. includens* population is undergoing demographic expansion. The results of the analysis (considering only individuals sampled in Brazil) are consistent with the history of diversification and demographic expansion observed in the Americas (i.e., similar haplotype network patterns). The demographic reconstruction traces the beginning of the *C. includens* population expansion back to the year 1,700, coinciding with the period of colonization of the Americas. However, the demographic reconstruction (i.e., Skyline analysis) must be cautiously interpreted, given that the genome‐wide mutation rate for this particular species is not known (El‐Shehawi & Elseehy, 2016; Keightley et al., 2014; Oppold & Pfenninger, 2017).

The pattern of low diversity and genetic structure observed with the mtDNA markers is congruent with nuclear markers and supports previous findings with ISSR markers (Palma et al., 2015). The lack of a clear genetic structure, despite the large geographic distances between sample sites, may be evidence of a recent range expansion, crop‐adapted lineages, source‐sink/meta‐population dynamics, or even *Wolbachia*‐associated lineages that could attenuate the signals of isolation by distance (IBD) (Forbes et al., 2017; Gonçalves et al., 2019; Hurst & Jiggins, 2005; Peterson & Denno, 1998). Large‐scale panmixia is not commonly reported and must not be considered the default pattern, given the limitations on the dispersal of most organisms that would prevent them from interbreeding without restrictions (Ritchie, Cousins, Cregeen, & Piertney, 2013; Teixeira, Serrão, & Arnaud‐Haond, 2012). Furthermore, in Brazil, we do not have reports of migratory behavior in Noctuidae moths that could increase the gene flow among geographically distant populations, but no conclusive study has been conducted to investigate this behavior in *C. includens*. However, our preliminary data of gossypol derived biomarker indicate that *C. includens* do not often engage in long‐distance dispersal in Brazil (Silva, 2020). Another potentially important variable is the antagonistic interaction between *C. includens* and the entomopathogenic fungus *Nomuraea rileyi* (Sosa‐Gómez, Delpin, Moscardi, & Nozaki, 2003). It is not known whether *N. rileyi* can significantly impact the population dynamics of *C. includens* on a large scale, affecting the effective population size and host interactions; however, fungicides might disrupt this interaction and favor *C. includens* in soybean systems as fungicide spraying increases to control soybean fungal diseases (Sosa‐Gómez et al., 2003).

Signals of differentiation by distance or geographic regions (i.e., *F*_ST_ grouping pattern) and by host (i.e., cotton and soybean) can be identified and indicate a process of differentiation driven by both genetic drift and positive selection. In our study, soybean fields were the predominant agricultural type in all landscapes, covering up to 50% of the total area in some cases (e.g., PRPI and BACO). The existing extensive soybean fields might be reducing genetic diversity in *C. includens*. If this is the case, then the levels of genetic diversity of *C. includens* may be affected by genetic drift, promoted by continuous processes of (re)colonization of soybean crops and rapid increases in population density within a short period. In a recent study, the number of moths captured by light traps in farmland in the Brazilian Cerrado was positively correlated with the presence and phenological stage of local soybean crops, where the period of high moth density in the area lasted only 60 days (Santos et al., 2017). This would indicate an abrupt population decrease, followed by a complete absence of insects from the area for a prolonged period of time. Depending on the scale of this phenomenon, the wide fluctuation in the number of insects can contribute to genetic drift and stochastic loss of genetic diversity. Furthermore, the number of individuals of *C. includens* has decreased in recent years at several sites (not to be confused with effective population size), along with the continuing adoption of *Bt* soybeans in Brazil (Santos et al., 2017). This effect was not captured in our study, which showed only signs of population demographic expansion, and continued monitoring of natural populations is necessary to measure the impact of *Bt* soybean crops on the effective population size of *C. includens*. Our data suggested, however, that the composition of the agricultural landscape, soybean and cotton in particular, is shaping the pattern of genetic diversity in *C. includens*. Therefore, a study of the temporal variation or meta‐population could offer additional insights into how seasonal gene flow and genetic drift might be affecting the process of natural selection in this species.

### 4.1 Natural selection and local adaptation

We have found compelling pieces of evidence that natural selection is shaping populations of *C. includens* in Brazil. These adaptive processes can be linked to the recent change in the pest status of *C. includens*. The insects are widely distributed and therefore experience environmental and habitat‐associated factors differently at different locations. Hence, both abiotic factors such as temperature and humidity, and biotic factors such as host plants can be putative drivers of the evolutionary process through natural selection. Based on the selection screenings, landscape composition seems to be a more important driver of the adaptive process than climate conditions.

Inevitably, extensive monoculture areas will not only favor the performance of certain herbivores but impose strong selection pressures on the populations of pest insects (Via, 1990; Wetzel, Kharouba, Robinson, Holyoak, & Karban, 2016). In addition to the extensive areas and the continuous succession of suitable hosts, soybean fields in the southwest and southern regions are cultivated in very intensive management systems with high input of fertilizers, reduced plant spacing, short plant cycles, and in recent years, the use of *Bt* crops. Specifically, *C. includens* is still very sensitive to *Bt* crops and has different levels of resistance to the different pesticides used on soybean and cotton, although no control failures have been reported (Bernardi et al., 2012; Stacke et al., 2019; Yano et al., 2016). However, this scenario can become more problematic when we consider the advance of agricultural frontiers into northern Brazil, increasing the soybean‐producing area even further. Considering the rapid habitat transformations in the Cerrado region in recent decades, local adaptation is an expected response for a population under such intensive selective forces in highly homogeneous areas (Mopper & Strauss, 1998). The greatest concern is that, once present, resistance to *Bt* or insecticides would spread quickly through the population. Among the proteins that may be associated with the management of pest‐insect resistance and host adaptation, a UDP‐glycosyltransferase protein (detected by LFMM and Lositan) has been detected (Kaplanoglu, Chapman, Scott, & Donly, 2017; Krempl et al., 2016). Further functional analysis and continued resistance monitoring may help to assess whether resistance is developing and spreading.

### 4.2 Host selection and agricultural landscapes

Understanding how genetic diversity is distributed over the range of a species is essential to study patterns of gene flow and to assess adaptive trends. Knowledge of the genetic structure of populations will enable us to more reliably infer which biotic and abiotic factors may be responsible for gene‐pool isolation or the lack of it (Avise, 1992; Slatkin, 1987). Habitat patchiness and host plant selection and distribution can counteract the effect of gene flow, producing genetic structure at fine spatial scales (Mopper, 1996). The evolution of host‐specialized races within polyphagous species is often reported (Forbes et al., 2017; Mopper, 1996; Thompson, 1988; Wood, Tilmon, Shantz, Harris, & Pesek, 1999). The formation of sympatric races in pest populations is not well understood, but extensive crop fields impose strong selection pressures on herbivory‐related adaptations, causing evolutionary divergence at fine scales (Carroll & Boyd, 1992; Feder et al., 1994; Gouin et al., 2017; Via, 1991). It is difficult to predict whether the differentiation by host reported here will increase or will be eroded by gene flow in the long term, particularly in unstable environments such as the agricultural systems. Moreover, the difference between insects collected from cotton and soybean plants was evident in only one of the two sampling locations (BACOC), for which the Blast2GO results revealed that feeding behavior associated with aminotransferase, amidases, carboxypeptidase, and carboxylase might be changing. BACOC had a much less diverse landscape, where cotton crops cover up to 14% of the land, and soybean was absent during the cotton‐growing period, whereas MTCVC had a more diverse landscape, where cotton covered only 5% of the total area, sharing the landscape with soybean fields. The smaller proportion of cotton in MTCVC, the greater degree of fragmentation (smaller patch sizes), and the presence of soybean could explain the different outcomes regarding the natural selection process in *C. includens* when cotton plants were the main driver. Further studies are needed to reinforce the evidence of host‐related race formation and the functional importance of the candidate genes listed here.

## 5 CONCLUSIONS

Both the mitochondrial and SNP markers revealed a pattern of low genetic diversity and a weak or absent genetic structure in *C. includens* in Brazil. This pattern could be attributed to recurrent evolutionary processes such as gene flow, or to a historical process associated with demographic expansion and host‐related adaptations. Analyses of the landscape composition suggested that the increase in the soybean crop area is likely important for the process of local adaptation, in particular in relation to host‐specific adaptations. Landscape composition and configuration may explain the still‐incipient differentiation between the hosts; and no effect of climate variables was detected. Our results reinforced the general recommendation for polyculture and for more diverse agroecosystems as strategies to prevent the rapid evolution of undesirable traits. Polyculture including one or more nonhost plants often limits the total number of pest insects relative to the numbers in monoculture systems, by reducing food resources and offering more opportunities for antagonistic ecological interactions with natural enemies, for instance (Risch 1981).

The present data increase our understanding of how the host range and future problems of *Bt* and pesticide resistance could affect *C. includens*. Understanding how adaptive processes shape the evolutionary trajectory of pest populations will not prevent the ongoing transformation of natural populations, but can guide us in a search for better resistance monitoring and crop‐succession management over time.

## CONFLICT OF INTEREST

None declared.

## AUTHORS’ CONTRIBUTION

ASC, CSS, EMGC, GH, PMD, and RAC conceived and designed the study. CSS collected the data. ASC, CSS, and EMGC analyzed the data. ASC, PMD, and RAC provided reagents, analytical tools, and financial support. ASC, CSS, and EMGC wrote the manuscript. All authors read, corrected, and approved the manuscript.

## Supporting information

Appendix S1‐S7

## Data Availability

Detailed information regarding the parameter selection in stacks, data curation, haplotype information, and landscape descriptions is included in the supplementary material. Raw fasta files of Illumina sequences were included in the SRA‐NCBI repository (PRJNA576576).
