# Supplementary material for: Population expansion and genomic adaptation to agricultural environments of the soybean looper, Chrysodeixis includens
Source: Evol Appl. 2020 Apr 19;13(8):2071–85. doi: 10.1111/eva.12966 (PMC7463353; doi:10.1111/eva.12966)
Supplement: Supplementary file 1 — Appendix S1‐S7 [file EVA-13-2071-s001.docx]

**Evolutionary Applications**

**Rapid expansion and genomic adaptation to agricultural environments of soybean looper, *Chrysodeixis includens***

**Appendix S1.** Measures of genetic diversity for *Chrysodeixis includens* (Lepidoptera: Noctuidae) based on two concatenated mitochondrial genes (COI–COII).

| **Location (City, State)** | **Code** | **Host** | **mtDNA haplotypes (*n*)*** | **Haplotype diversity (Hd)** | **Nucleotide diversity (π)** | **Latitude (S)** | **Longitude (W)** |
| --- | --- | --- | --- | --- | --- | --- | --- |
| *Atlantic Forest biome* | | | *H1(10), H2, H3, H4, H5* | *0.505* | *0.00059* |  | |
| Coxilha, RS | RSCO | Soybean | H1(3) | 0.000 | 0.00000 | 28°10’54’’ | 52°44’46’’ |
| Itapira, SC | SCIT | Soybean | H1(2), H2 | 0.667 | 0.00055 | 27°8’43’’ | 53°43’53’’ |
| Pitanga, PR | PRPI | Soybean | H1(2), H3 | 0.667 | 0.00055 | 24°17’23’’ | 52°34’16’’ |
| Casa Branca, SP | SPCB | Soybean | H1(3), H4, H5 | 0.700 | 0.00098 | 21°43’34’’ | 47°09’09’’ |
| *Cerrado biome* | | | *H1(19), H2(2) H6(3), H7, H8, H9, H10, H11, H12(2)* | *0.622* | *0.00067* |  | |
| Araguari, MG | MGAR | Soybean | H1(3), H6, H7 | 0.700 | 0.00066 | 18°59’23’’ | 47°33’52’’ |
| Sete Lagoas, MG | MGSL | Soybean | H1(2), H6 | 0.667 | 0.00055 | 19°27’51’’ | 44°10’38’’ |
| Campo Grande, MS | MSCG | Soybean | H1(4), H8 | 0.400 | 0.00033 | 20°42’31’’ | 54°30’03’’ |
| Chapadão do Sul, MS | MSCS | Cotton | H1, H2, H9 | 1.000 | 0.00164 | 18°44’54’’ | 52°35’44’’ |
| Mineiros, GO | GOMI | Soybean | H1(5), H2 | 0.333 | 0.00027 | 17°37’43’’ | 52°36’52’’ |
| Campo Verde, MT | MTCV | Cotton | H1(2), H6, H10 | 0.833 | 0.00082 | 15°36’40’’ | 55°14’05’’ |
| Campo Verde, MT | MTCV | Soybean | H1(2), H11, H12(2) | 0.800 | 0.00082 | 15°25’34’’ | 54°48’05’’ |
| *Caatinga biome* | | | *H1 (16)* | *0.000* | *0.00000* |  | |
| Correntina, BA | BACO | Soybean | H1(6) | 0.000 | 0.00000 | 11°49’21’’ | 46°10’52’’ |
| Junqueiro, AL | ALJU | Bean | H1(2) | 0.000 | 0.00000 | 9°51’58’’ | 36°25’29’’ |
| Russas, CE | CERU | Bean | H1(6) | 0.000 | 0.00000 | 4°55’28’’ | 38°00’14’’ |
| Teresina, PI | PITE | Bean | H1(2) | 0.000 | 0.00000 | 5°02’21’’ | 42°47’22’’ |
| *All locations* |  |  | *H1(45), H2(3), H3, H4, H5, H6(3), H7, H8, H9, H10, H11, H12(2)* | *0.455* | *0.00048* |  |  |

* The frequency of each haplotype among the samples (n>1) is given in parentheses

**Appendix S2.** COI sequence network of *Chrysodeixis includens* from the Americas.

To test if *C. includens* underwent a recent demographic expansion, we performed two additional analyses of genealogical relationships with datasets constructed with the COI sequences obtained in this study and downloaded from NCBI (as of June 12, 2018). The dataset_S1 includes COI sequences of *C. includens* from Brazil (this study, *n* = 61) and additional COI sequences from Brazil (*n* = 20), Costa Rica (*n* = 38), the USA (*n* = 18), and Canada (*n* = 2). Here, we characterized the haplotypes using DnaSP ver. 5 (Librado & Rozas, 2009) (**Table S2**), and their genealogical relationships are shown in the haplotype median-joining network, generated by using PopART software (**Figure S2**). The star-like topology of the haplotype network **(Figure S2)** and low haplotype diversity in the mitochondrial COI sequences **(Table S2)** from insects collected in South, Central, and North America support the hypothesis of a rapid and recent demographic and spatial expansion of *C. includens*.

**
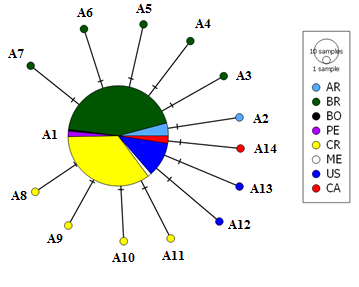
**

**Figure S2.** Parsimony network of mtDNA haplotypes of *Chrysodeixis includens*, based on a 522-bp fragment of the COI gene. Numbers correspond to the haplotype numbers in **Table S2**. Circle size is proportional to haplotype frequency in the population sampled (see scale at upper right). Hatch marks represent the number of mutation steps separating two haplotypes and colors indicate the origin of the sequences. Argentina (AR), Brazil (BR), Bolivia (BR), Peru (PE), Costa Rica (CR), Mexico (ME), the United States (USA), and Canada (CA).

**Table S2.** Number of haplotypes of *C. includens* (Lepidoptera: Noctuidae) collected in GenBank in eight different countries from the American continent based on a 522-bp fragment of the COI gene. The frequency of each haplotype among the samples (*n*>1) is given in parentheses.

| **Location** | **Haplotypes (n)*** | **Bold system** |
| --- | --- | --- |
| Argentina | A1(7), A2 | ARMOT129-12; [**ARMOT187-12**](http://www.boldsystems.org/index.php/Public_RecordView?processid=ARMOT187-12); [**ECPD519-14**](http://www.boldsystems.org/index.php/Public_RecordView?processid=ECPD519-14)**;**  [**LEPPA1129-14**](http://www.boldsystems.org/index.php/Public_RecordView?processid=LEPPA1129-14); [**LEPPA1141-14**](http://www.boldsystems.org/index.php/Public_RecordView?processid=LEPPA1141-14); [**MOTAR192-12**](http://www.boldsystems.org/index.php/Public_RecordView?processid=MOTAR192-12); [**MOTAR197-12**](http://www.boldsystems.org/index.php/Public_RecordView?processid=MOTAR197-12); [**MOTAR206-12**](http://www.boldsystems.org/index.php/Public_RecordView?processid=MOTAR206-12) |
| Brazil | A1(74), A3, A4, A5, A6, A7 | [**GBGL12665-13**](http://www.boldsystems.org/index.php/Public_RecordView?processid=GBGL12665-13)**;** [**GBGL12666-13**](http://www.boldsystems.org/index.php/Public_RecordView?processid=GBGL12666-13)**;** [**GBGL12667-13**](http://www.boldsystems.org/index.php/Public_RecordView?processid=GBGL12667-13)**;**  [**GBGL12668-13**](http://www.boldsystems.org/index.php/Public_RecordView?processid=GBGL12668-13)**;** [**GBGL12669-13**](http://www.boldsystems.org/index.php/Public_RecordView?processid=GBGL12669-13)**;** [**GBGL12670-13**](http://www.boldsystems.org/index.php/Public_RecordView?processid=GBGL12670-13)**;**  [**GBGL12671-13**](http://www.boldsystems.org/index.php/Public_RecordView?processid=GBGL12671-13)**;** [**GBGL12672-13**](http://www.boldsystems.org/index.php/Public_RecordView?processid=GBGL12672-13)**;** [**GBGL12673-13**](http://www.boldsystems.org/index.php/Public_RecordView?processid=GBGL12673-13)**;** [**GBGL12674-13**](http://www.boldsystems.org/index.php/Public_RecordView?processid=GBGL12674-13)**;** [**GBGL12675-13**](http://www.boldsystems.org/index.php/Public_RecordView?processid=GBGL12675-13)**;** [**GBGL12676-13**](http://www.boldsystems.org/index.php/Public_RecordView?processid=GBGL12676-13)**;**  [**LEMMZ135-10**](http://www.boldsystems.org/index.php/Public_RecordView?processid=LEMMZ135-10)**;** [**LEMMZ136-10**](http://www.boldsystems.org/index.php/Public_RecordView?processid=LEMMZ136-10)**;** [**LEMMZ137-10**](http://www.boldsystems.org/index.php/Public_RecordView?processid=LEMMZ137-10)**;**  [**LEMMZ138-10**](http://www.boldsystems.org/index.php/Public_RecordView?processid=LEMMZ138-10)**;** [**LEMMZ374-10**](http://www.boldsystems.org/index.php/Public_RecordView?processid=LEMMZ374-10)**;** [**LEMMZ375-10**](http://www.boldsystems.org/index.php/Public_RecordView?processid=LEMMZ375-10)**;** |
| Bolivia | A1 | [**NOCJB053-08**](http://www.boldsystems.org/index.php/Public_RecordView?processid=NOCJB053-08)**;** |
| Peru | A1(3) | [**LNAUP106-13**](http://www.boldsystems.org/index.php/Public_RecordView?processid=LNAUP106-13)**;** [**LNAUP107-13**](http://www.boldsystems.org/index.php/Public_RecordView?processid=LNAUP107-13)**;** [**NOCJB052-08**](http://www.boldsystems.org/index.php/Public_RecordView?processid=NOCJB052-08) |
| Costa Rica | A1(59), A8, A9, A10, A11 | [**BLPAA312-06**](http://www.boldsystems.org/index.php/Public_RecordView?processid=BLPAA312-06)**;** [**BLPAA727-06**](http://www.boldsystems.org/index.php/Public_RecordView?processid=BLPAA727-06)**;** [**BLPAA848-06**](http://www.boldsystems.org/index.php/Public_RecordView?processid=BLPAA848-06)**;**  [**BLPAB056-06**](http://www.boldsystems.org/index.php/Public_RecordView?processid=BLPAB056-06)**;** [**BLPAB057-06**](http://www.boldsystems.org/index.php/Public_RecordView?processid=BLPAB057-06)**;** [**BLPAB093-06**](http://www.boldsystems.org/index.php/Public_RecordView?processid=BLPAB093-06)**;**  [**BLPAF765-07**](http://www.boldsystems.org/index.php/Public_RecordView?processid=BLPAF765-07)**;** [**BLPAF924-07**](http://www.boldsystems.org/index.php/Public_RecordView?processid=BLPAF924-07)**;** [**BLPCC493-08**](http://www.boldsystems.org/index.php/Public_RecordView?processid=BLPCC493-08)**;** [**BLPCC493-08**](http://www.boldsystems.org/index.php/Public_RecordView?processid=BLPCC493-08)**;** [**BLPCD637-08**](http://www.boldsystems.org/index.php/Public_RecordView?processid=BLPCD637-08)**;** [**BLPDK1887-09**](http://www.boldsystems.org/index.php/Public_RecordView?processid=BLPDK1887-09)**;**  [**BLPDU205-11**](http://www.boldsystems.org/index.php/Public_RecordView?processid=BLPDU205-11)**;** [**BLPDU206-11**](http://www.boldsystems.org/index.php/Public_RecordView?processid=BLPDU206-11)**;** [**BLPDU207-11**](http://www.boldsystems.org/index.php/Public_RecordView?processid=BLPDU207-11)**;**  [**BLPDU520-11**](http://www.boldsystems.org/index.php/Public_RecordView?processid=BLPDU520-11)**;** [**BLPDU802-11**](http://www.boldsystems.org/index.php/Public_RecordView?processid=BLPDU802-11)**;** [**BLPDV008-11**](http://www.boldsystems.org/index.php/Public_RecordView?processid=BLPDV008-11)**;**  [**BLPDV009-11**](http://www.boldsystems.org/index.php/Public_RecordView?processid=BLPDV009-11)**;**[**LOCRA264-06**](http://www.boldsystems.org/index.php/Public_RecordView?processid=LOCRA264-06)**;** [**MHAUB696-05**](http://www.boldsystems.org/index.php/Public_RecordView?processid=MHAUB696-05)**;** [**MHAUB697-05**](http://www.boldsystems.org/index.php/Public_RecordView?processid=MHAUB697-05)**;** [**MHAUB698-05**](http://www.boldsystems.org/index.php/Public_RecordView?processid=MHAUB698-05)**;** [**MHAUB699-05**](http://www.boldsystems.org/index.php/Public_RecordView?processid=MHAUB699-05)**;**  [**MHAUB700-05**](http://www.boldsystems.org/index.php/Public_RecordView?processid=MHAUB700-05)**;** [**MHAUB701-05**](http://www.boldsystems.org/index.php/Public_RecordView?processid=MHAUB701-05)**;** [**MHAUB702-05**](http://www.boldsystems.org/index.php/Public_RecordView?processid=MHAUB702-05)**;** [**MHAUB703-05**](http://www.boldsystems.org/index.php/Public_RecordView?processid=MHAUB703-05)**;** [**MHAUB704-05**](http://www.boldsystems.org/index.php/Public_RecordView?processid=MHAUB704-05)**;** [**MHAUB705-05**](http://www.boldsystems.org/index.php/Public_RecordView?processid=MHAUB705-05)**;**  [**MHAUG100-07**](http://www.boldsystems.org/index.php/Public_RecordView?processid=MHAUG100-07)**;** [**MHAUG678-07**](http://www.boldsystems.org/index.php/Public_RecordView?processid=MHAUG678-07)**;** [**MHAUG679-07**](http://www.boldsystems.org/index.php/Public_RecordView?processid=MHAUG679-07)**;**  [**MHAUG680-07**](http://www.boldsystems.org/index.php/Public_RecordView?processid=MHAUG680-07)**;**  [**MHAUG681-07**](http://www.boldsystems.org/index.php/Public_RecordView?processid=MHAUG681-07)**;** [**MHAUG682-07**](http://www.boldsystems.org/index.php/Public_RecordView?processid=MHAUG682-07)**;**  [**MHAUG683-07**](http://www.boldsystems.org/index.php/Public_RecordView?processid=MHAUG683-07)**;** [**MHMXA782-06**](http://www.boldsystems.org/index.php/Public_RecordView?processid=MHMXA782-06)**;** [**MHMXA818-06**](http://www.boldsystems.org/index.php/Public_RecordView?processid=MHMXA818-06)**;** [**MHMXE344-06**](http://www.boldsystems.org/index.php/Public_RecordView?processid=MHMXE344-06)**;** [**MHMXI418-07**](http://www.boldsystems.org/index.php/Public_RecordView?processid=MHMXI418-07)**;**  [**MHMXM101-07**](http://www.boldsystems.org/index.php/Public_RecordView?processid=MHMXM101-07)**;**  [**MHMXM170-07**](http://www.boldsystems.org/index.php/Public_RecordView?processid=MHMXM170-07)**;** [**MHMXM171-07**](http://www.boldsystems.org/index.php/Public_RecordView?processid=MHMXM171-07)**;** [**MHMXM172-07**](http://www.boldsystems.org/index.php/Public_RecordView?processid=MHMXM172-07)**;** [**MHMXM173-07**](http://www.boldsystems.org/index.php/Public_RecordView?processid=MHMXM173-07)**;** [**MHMXM174-07**](http://www.boldsystems.org/index.php/Public_RecordView?processid=MHMXM174-07)**;** [**MHMXM175-07**](http://www.boldsystems.org/index.php/Public_RecordView?processid=MHMXM175-07)**;** [**MHMYC2167-09**](http://www.boldsystems.org/index.php/Public_RecordView?processid=MHMYC2167-09)**;** [**MHMYH812-10**](http://www.boldsystems.org/index.php/Public_RecordView?processid=MHMYH812-10)**;** [**MHMYH813-10**](http://www.boldsystems.org/index.php/Public_RecordView?processid=MHMYH813-10)**;** [**MHMYL3084-11**](http://www.boldsystems.org/index.php/Public_RecordView?processid=MHMYL3084-11)**;** [**MHMYL3269-11**](http://www.boldsystems.org/index.php/Public_RecordView?processid=MHMYL3269-11)**;** [**MHMYL3281-11**](http://www.boldsystems.org/index.php/Public_RecordView?processid=MHMYL3281-11)**;**  [**MHMYL3282-11**](http://www.boldsystems.org/index.php/Public_RecordView?processid=MHMYL3282-11)**;** [**MHMYL3283-11**](http://www.boldsystems.org/index.php/Public_RecordView?processid=MHMYL3283-11)**;** [**MHMYL3411-11**](http://www.boldsystems.org/index.php/Public_RecordView?processid=MHMYL3411-11)**;**[**MHMYL3504-11**](http://www.boldsystems.org/index.php/Public_RecordView?processid=MHMYL3504-11)**;** [**MHMYM006-11**](http://www.boldsystems.org/index.php/Public_RecordView?processid=MHMYM006-11)**;** [**MHMYM085-11**](http://www.boldsystems.org/index.php/Public_RecordView?processid=MHMYM085-11)**;** [**MHMYM140-11**](http://www.boldsystems.org/index.php/Public_RecordView?processid=MHMYM140-11)**;** [**MHMYO207-11**](http://www.boldsystems.org/index.php/Public_RecordView?processid=MHMYO207-11)**;** |
| Mexico | A1(2) | [**GMMAH010-15**](http://www.boldsystems.org/index.php/Public_RecordView?processid=GMMAH010-15)**;** [**GMMCS002-15**](http://www.boldsystems.org/index.php/Public_RecordView?processid=GMMCS002-15)**;**  [**LPYPB275-08**](http://www.boldsystems.org/index.php/Public_RecordView?processid=LPYPB275-08) |
| USA | A1(19), A12, A13 | [**LGSMG611-07**](http://www.boldsystems.org/index.php/Public_RecordView?processid=LGSMG611-07)**;** [**LGSMG612-07**](http://www.boldsystems.org/index.php/Public_RecordView?processid=LGSMG612-07)**;** [**LGSMG613-07**](http://www.boldsystems.org/index.php/Public_RecordView?processid=LGSMG613-07)**;**  [**LILLA916-11**](http://www.boldsystems.org/index.php/Public_RecordView?processid=LILLA916-11)**;** [**LILLA958-11**](http://www.boldsystems.org/index.php/Public_RecordView?processid=LILLA958-11)**;** [**LILLB064-11**](http://www.boldsystems.org/index.php/Public_RecordView?processid=LILLB064-11)**;**  [**LNC849-06**](http://www.boldsystems.org/index.php/Public_RecordView?processid=LNC849-06)**;** [**LNCB071-06**](http://www.boldsystems.org/index.php/Public_RecordView?processid=LNCB071-06)**;** [**LOCBF3743-14**](http://www.boldsystems.org/index.php/Public_RecordView?processid=LOCBF3743-14)**;** [**LOCBF4241-14**](http://www.boldsystems.org/index.php/Public_RecordView?processid=LOCBF4241-14)**;** [**LOCBF4242-14**](http://www.boldsystems.org/index.php/Public_RecordView?processid=LOCBF4242-14)**;** [**LOFLB770-06**](http://www.boldsystems.org/index.php/Public_RecordView?processid=LOFLB770-06)**;**  [**LPOKA1032-09**](http://www.boldsystems.org/index.php/Public_RecordView?processid=LPOKA1032-09)**;** [**LPOKA343-08**](http://www.boldsystems.org/index.php/Public_RecordView?processid=LPOKA343-08)**;** [**LPOKA567-09**](http://www.boldsystems.org/index.php/Public_RecordView?processid=LPOKA567-09)**;**  [**LPOKA628-09**](http://www.boldsystems.org/index.php/Public_RecordView?processid=LPOKA628-09)**;**  [**LPOKA661-09**](http://www.boldsystems.org/index.php/Public_RecordView?processid=LPOKA661-09)**;** [**LPOKD407-09**](http://www.boldsystems.org/index.php/Public_RecordView?processid=LPOKD407-09)**;**  [**LPOKD427-09**](http://www.boldsystems.org/index.php/Public_RecordView?processid=LPOKD427-09)**;** [**LPOKD432-09**](http://www.boldsystems.org/index.php/Public_RecordView?processid=LPOKD432-09)**;** [**LPOKD449-09**](http://www.boldsystems.org/index.php/Public_RecordView?processid=LPOKD449-09)**;** |
| Canada | A1(4), A14 | [**RDLQ426-07**](http://www.boldsystems.org/index.php/Public_RecordView?processid=RDLQ426-07)**;** [**SMTPO4162-15**](http://www.boldsystems.org/index.php/Public_RecordView?processid=SMTPO4162-15)**;** [**SMTPR191-16**](http://www.boldsystems.org/index.php/Public_RecordView?processid=SMTPR191-16)**;**  [**SMTPR7089-16**](http://www.boldsystems.org/index.php/Public_RecordView?processid=SMTPR7089-16)**;** [**XAB463-04**](http://www.boldsystems.org/index.php/Public_RecordView?processid=XAB463-04)**;** [**XAH608-05**](http://www.boldsystems.org/index.php/Public_RecordView?processid=XAH608-05)**;** |

*Reference:*

Librado, P., & Rozas, J. (2009). DnaSP v5: a software for comprehensive analysis of DNA polymorphism data. Bioinformatics, 25(11), 1451–1452.

**Appendix S3.** *Single-nucleotide polymorphism (SNP) discovery and data summary*

The GBS library was constructed by the EcoMol Consultoria e Projetos (Piracicaba, Brazil) following the protocol described by Elshire et al. (2011) with modifications. Initially, the DNA cleavage of samples with the PstI and ApekI enzymes was tested, following the manufacturer’s protocol (New England BioLabs®). The PstI enzyme generated more uniform and homogeneously distributed fragments. The adapters were previously designed using the GBS Barcode Generator tool (Deena Bioinformatics), taking into consideration the barcode sequence, in order to maximize the balance of the bases at each position in the defined set. 100 ng of DNA was cleaved with 0.2 μL PstI (10 U/μL) at 37 °C for 2 h. Next, the enzyme was deactivated at 85 °C for 20 s and the samples were dehydrated. To perform the ligation reaction, we rehydrated all samples in 6 μL of adapter solutions and incubated them at 22 °C for 2 h in a binding mix with T4 DNA ligase (New England BioLabs®). All samples were pooled and purified using the QIAquick PCR Purification Kit® (Qiagen). PCR amplification was conducted using specific primers for sequencing, using the Illumina platform. PCR purification was carried out using the Agencourt AMPure XP PCR purification kit® (Beckman Coulter). PCR products were quantified by quantitative PCR, using the KAPA Library Quantification Kit (KAPA Biosystems). The library was clustered using a TruSeq SR Cluster Kit v3-cBot-HS on the cBOT system (Illumina, San Diego) and sequenced at the Animal Genome Centre of ESALQ/USP, using the Illumina TruSeq SBS Kit v3-HS in an Illumina HiSeq2500 sequencer.

The two sequenced GBS libraries yielded a total of 784,309,732 reads. Of these, 696,980,827 reads were retained (~89%) after the demultiplexing and quality-filtering steps. Next, 315,667 loci were successfully genotyped with a mean coverage per sample of 106.3× (SD = 29.5×) and minimum and maximum of 14.0× and 182.0×, respectively. After missing data were filtered, 2,173 variant sites from 1,453 polymorphic loci were retained for further analysis.

**
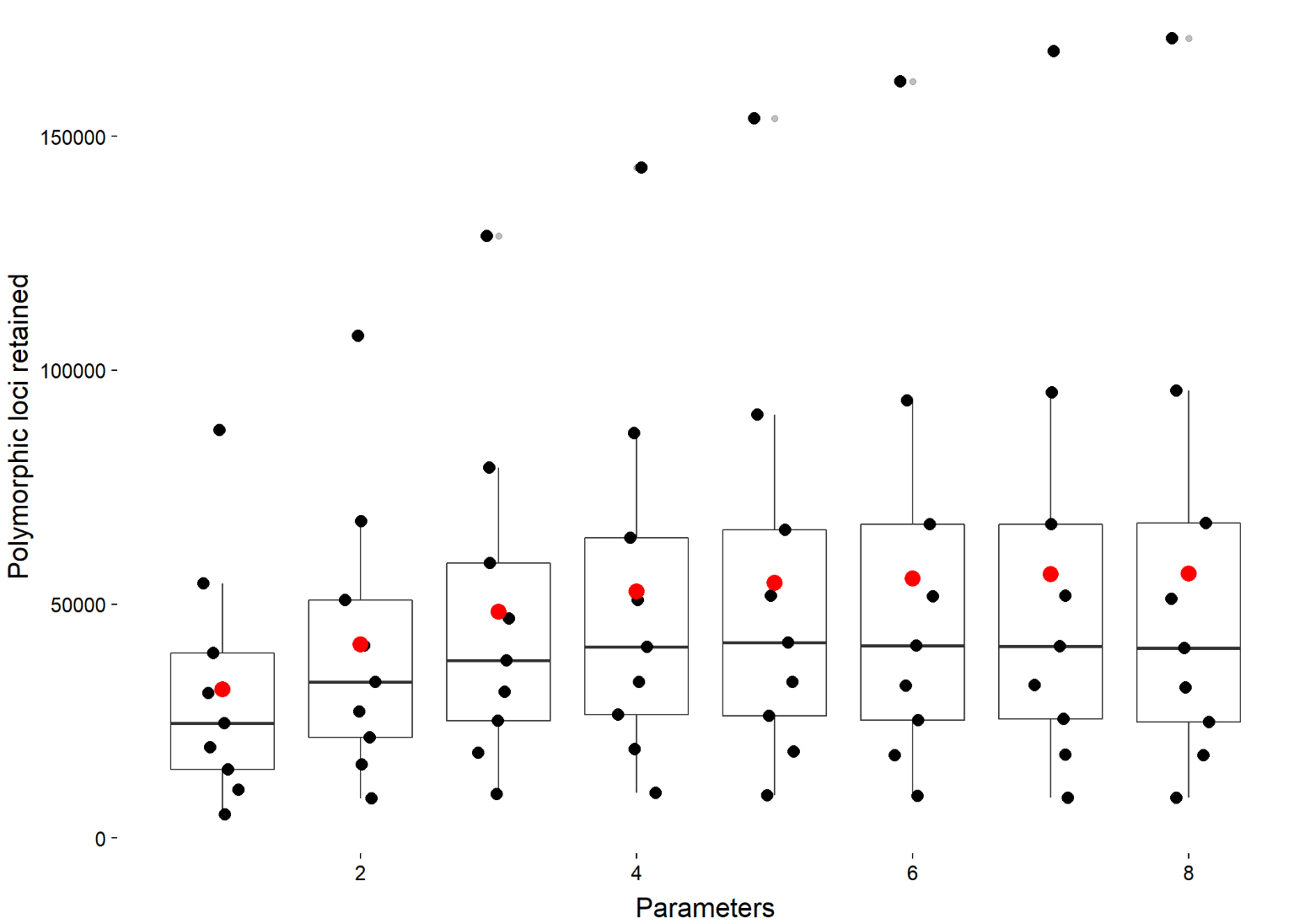
**

**Figure S3. Number of polymorphic loci retained in function of the distance allowed between two stacks (M).** The distribution of each value of M was generated by varying values of the minimum percentage of individuals in a population (-*r*) from 10% (*r10*) to 90% (*r90*). Red dot represents the mean. Mean values of M stabilize between M3 and M4.

**Appendix S4.** Analysis of molecular variance (AMOVA) for genetic structure of *C. includens* (Lepidoptera: Noctuidae) by collection local from Brazil based on cytochrome oxidase subunit I (COI) and cytochrome oxidase subunit II (COII) concatenated gene fragment sequences.

| Source of variation | *d.f.* | Variance component | Percentage variance | Fixation index  (*p*-value) |
| --- | --- | --- | --- | --- |
| Among populations | 14 | 0.0055 | 1.90 | Φ_ST_ = 0.019 (*p*=0.25) |
| Within populations | 46 | 0.2855 | 98.10 |  |
| Total | 60 | 0.2910 |  |  |

**Appendix S5. Landscape characterization**

This study consisted of the development of soil application maps, based on the collection of data from helicopters and a study of the landscape. Silveira (2015) stated that human beings have intensively altered the ecosystem and have tended to simplify it. The expansion of monoculture and the reduction of natural vegetation, according to the author, may have aggravated problems with agricultural pests. The environment that is altered by agricultural practices can pose geographical barriers to gene flow for some insects, and large amounts of resources for others. In this way, species of insects that are not pests under natural conditions become pests and cause significant crop damage. Finally, study of the landscape is important to assess the potential for dispersal of pests.

Silveira, F. A. O., Negreiros, D., Barbosa, N. P. U., Buisson, E., Carmo, F. F., Carstensen, D. W., et al. (2015). Ecology and evolution of plant diversity in the endangered campo rupestre: a neglected conservation priority. Plant Soil 403, 129–152.

**Figure S5A**. Land use and cover map obtained from a 25-km radius (1.96×10^9^ m^2^) of the sampled location in Russas, Ceará (CERU).


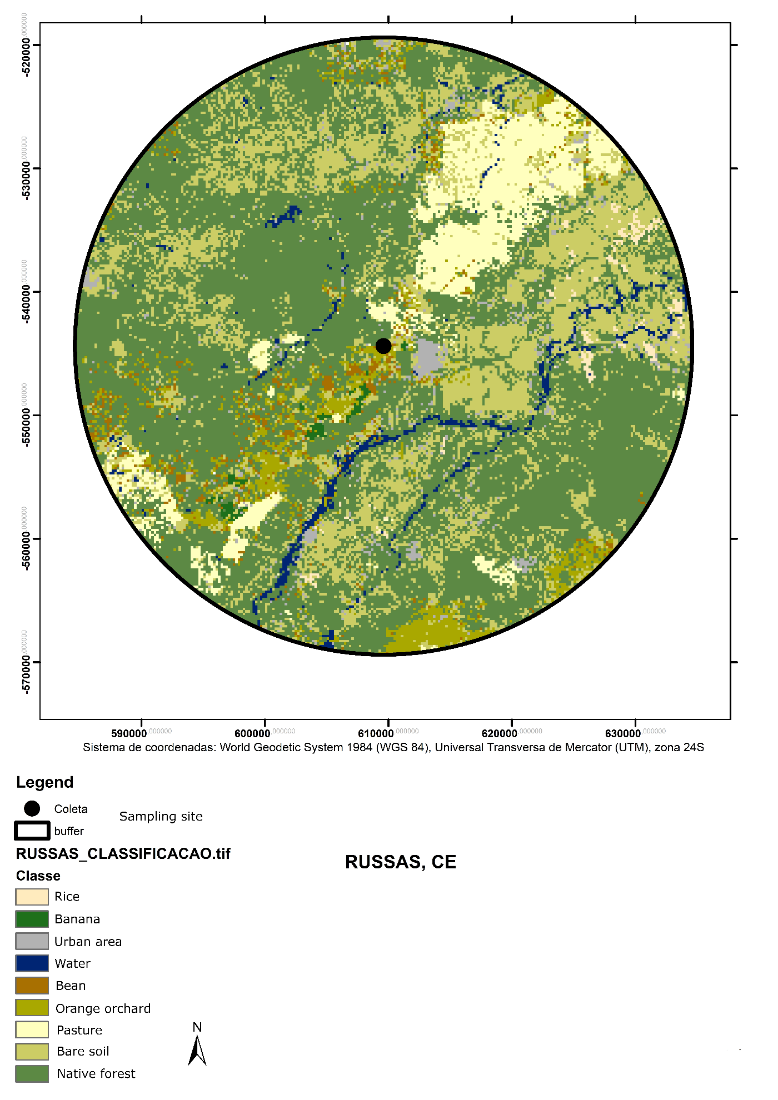


**Table S5A**. Landscape information by attributes present in a 25-km radius (1.96×10^9^ m^2^) in Russas, Ceará (CERU).

| Class | Land cover  (m^2^) | Landscape proportion | Edge length (m) | Edge density | Number of patches | Mean patch area (m^2^) |
| --- | --- | --- | --- | --- | --- | --- |
| Bare soil | 491640000 | 0.196656 | 4520800 | 0.002302724 | 1328 | 370210.8434 |
| Native forest | 1079040000 | 0.431616 | 5057200 | 0.002575946 | 402 | 2684179.104 |
| Pasture | 164800000 | 0.06592 | 769600 | 0.000392005 | 77 | 2140259.74 |
| Orange orchard | 94880000 | 0.037952 | 1066800 | 0.000543387 | 546 | 173772.8938 |
| Water | 36520000 | 0.014608 | 426800 | 0.000217396 | 155 | 235612.9032 |
| Urban area | 43560000 | 0.017424 | 638800 | 0.00032538 | 539 | 80816.32653 |
| Bean | 33000000 | 0.0132 | 522000 | 0.000265887 | 429 | 76923.07692 |
| Banana | 6840000 | 0.002736 | 56800 | 2.89E-05 | 11 | 621818.1818 |
| Rice | 12960000 | 0.005184 | 148400 | 7.56E-05 | 61 | 212459.0164 |

**Figure S5B**. Land use and cover map obtained from a 25-km radius (1.96×10^9^ m^2^) of the sampled location in Teresina, PI (PITE).


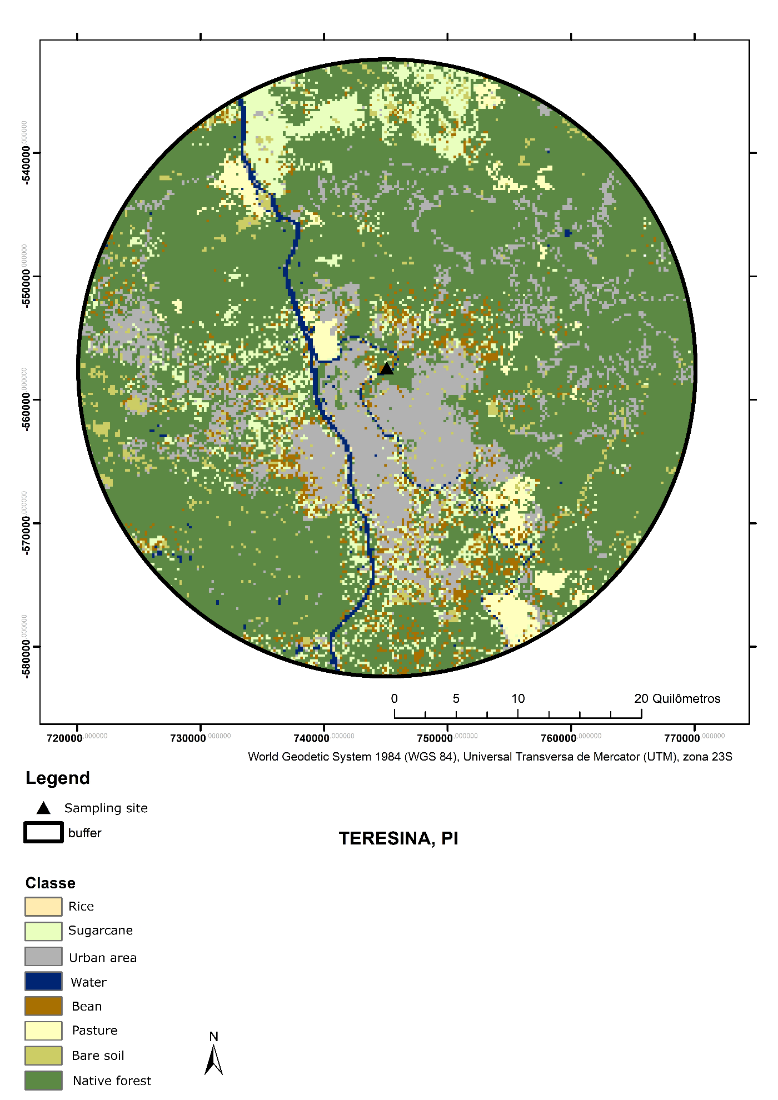


**Table S5B**. Landscape information by attributes present in 25 km radio (1.96×10^9^ m^2^) in Teresina, PI (PITE).

| Class | Land cover | Landscape Proportion | Edge length (m) | Edge density | Number of Patches | Mean patch area |
| --- | --- | --- | --- | --- | --- | --- |
| Bare soil | 64480000 | 0.025792 | 939600 | 0.000478626 | 703 | 91721.19488 |
| Native forest | 1275120000 | 0.510048 | 3982400 | 0.002028608 | 287 | 4442926.829 |
| Pasture | 51720000 | 0.020688 | 310400 | 0.000158116 | 42 | 1231428.571 |
| Water | 26720000 | 0.010688 | 288800 | 0.000147113 | 118 | 226440.678 |
| Sugarcane | 175160000 | 0.070064 | 1966000 | 0.001001467 | 952 | 183991.5966 |
| Urban area | 282480000 | 0.112992 | 2050800 | 0.001044664 | 549 | 514535.5191 |
| Bean | 86600000 | 0.03464 | 1342800 | 0.000684013 | 912 | 94956.14035 |
| Rice | 840000 | 0.000336 | 16000 | 8.15E-06 | 19 | 44210.52632 |

**Figure S5C.** Land use and cover map obtained from a 25-km radius (1.96×10^9^ m^2^) of the sampled location in Correntina, BA (BACOS).


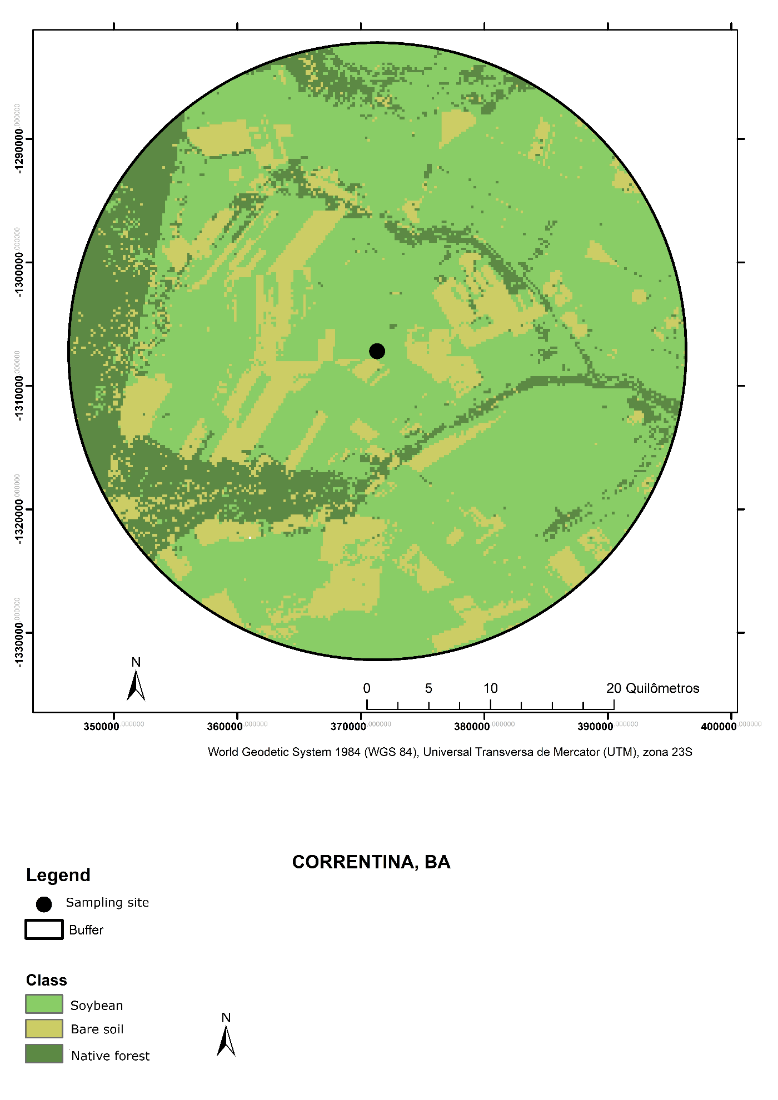


**Table S5C.** Landscape information by attributes present in 25 km radio (1.96×10^9^ m^2^) in Correntina, BA (BACOS).

| Class | Land cover | Landscape Proportion | Edge length (m) | Edge density | Number of Patches | Mean patch area |
| --- | --- | --- | --- | --- | --- | --- |
| Soybean | 1286800000 | 0.51472 | 2181200 | 0.001111 | 178 | 7229213.483 |
| Bare soil | 358960000 | 0.143584 | 1692000 | 0.000862 | 459 | 782047.9303 |
| Native forest | 317680000 | 0.127072 | 1547600 | 0.000788 | 322 | 986583.8509 |

**Figure S5D.** Land use and cover map obtained from a 25-km radius (1.96×10^9^ m^2^) of the sampled location in Correntina, Bahia (BACOC).


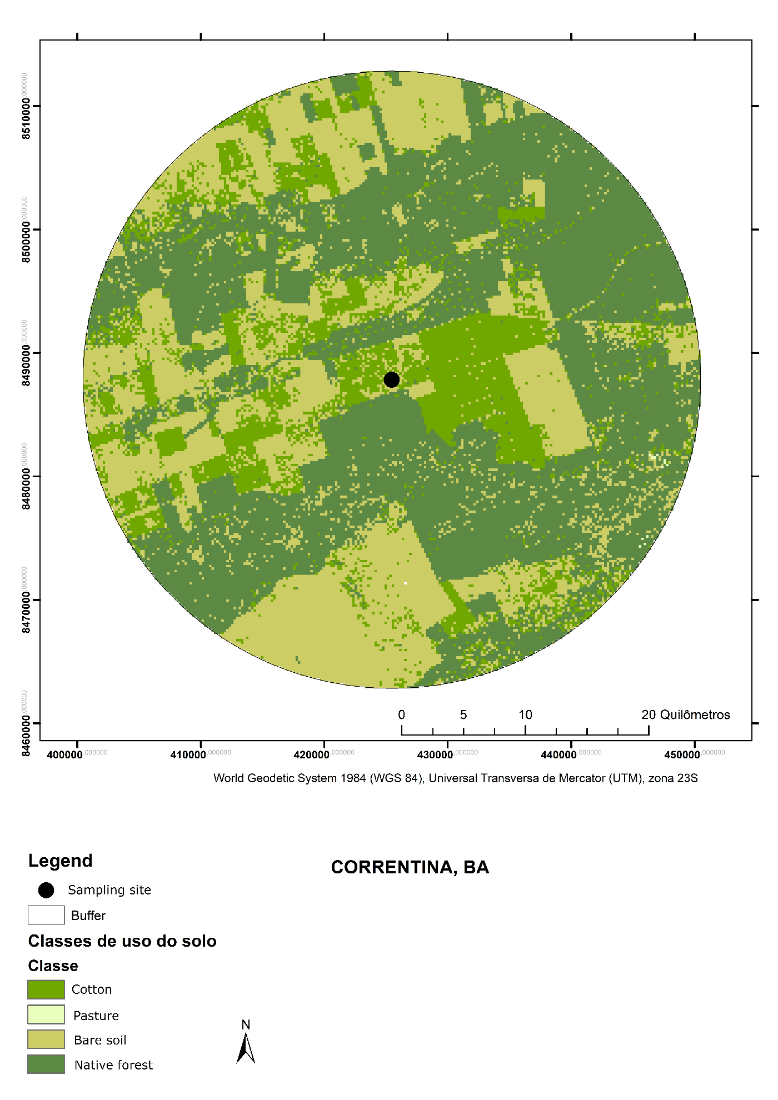


**Table S5D.** Landscape information by attributes present in 25 km radio (1.96×10^9^ m^2^) in Correntina, Bahia (BACOC).

| Class | Land cover | Landscape Proportion | Edge length (m) | Edge density | Number of Patches | Mean patch area |
| --- | --- | --- | --- | --- | --- | --- |
| Bare soil | 653640000 | 0.261456 | 3147600 | 0.001602811 | 939 | 696102.2364 |
| Native forest | 964400000 | 0.38576 | 2527200 | 0.001286893 | 203 | 4750738.916 |
| Pasture | 480000 | 0.000192 | 8800 | 4.48E-06 | 7 | 68571.42857 |
| Cotton | 345280000 | 0.138112 | 2440400 | 0.001242693 | 925 | 373275.6757 |

**Figure S5E.** Land use and cover map obtained from a 25-km radius (1.96×10^9^ m^2^) of the sampled location in Campo Verde, MT (MTCV).


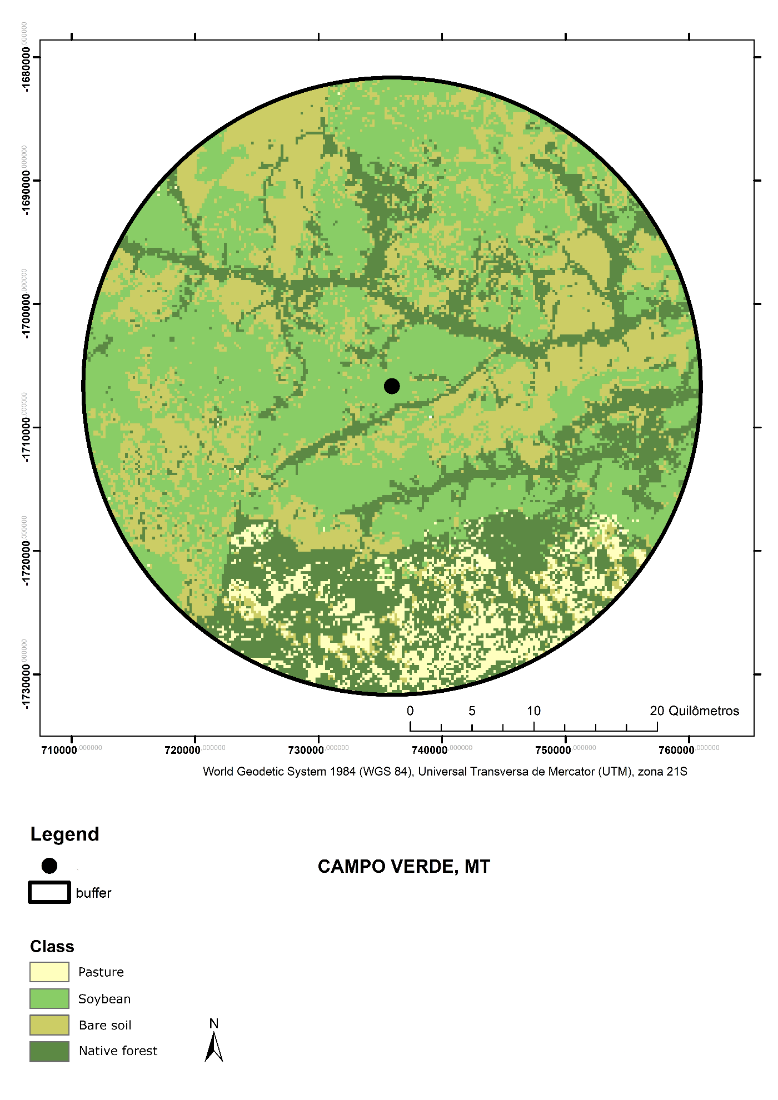


**Table S5E.** Landscape information by attributes present in 25 km radio (1.96×10^9^ m^2^) in Campo Verde, MT (MTCVS).

| Class | Land cover | Landscape Proportion | Edge length (m) | Edge density | Number of Patches | Mean patch area |
| --- | --- | --- | --- | --- | --- | --- |
| Soybean | 787720000 | 0.315088 | 3054000 | 0.00155575 | 388 | 2030206.186 |
| Bare soil | 553040000 | 0.221216 | 3336800 | 0.001699813 | 858 | 644568.7646 |
| Native forest | 504320000 | 0.201728 | 2879600 | 0.001466908 | 431 | 1170116.009 |
| Pasture | 117960000 | 0.047184 | 1007600 | 0.000513286 | 197 | 598781.7259 |

**Figure S5F.** Land use and cover map obtained from a 25-km radius (1.96×10^9^ m^2^) of the sampled location in Campo Verde, Mato Grosso (MTCVC).


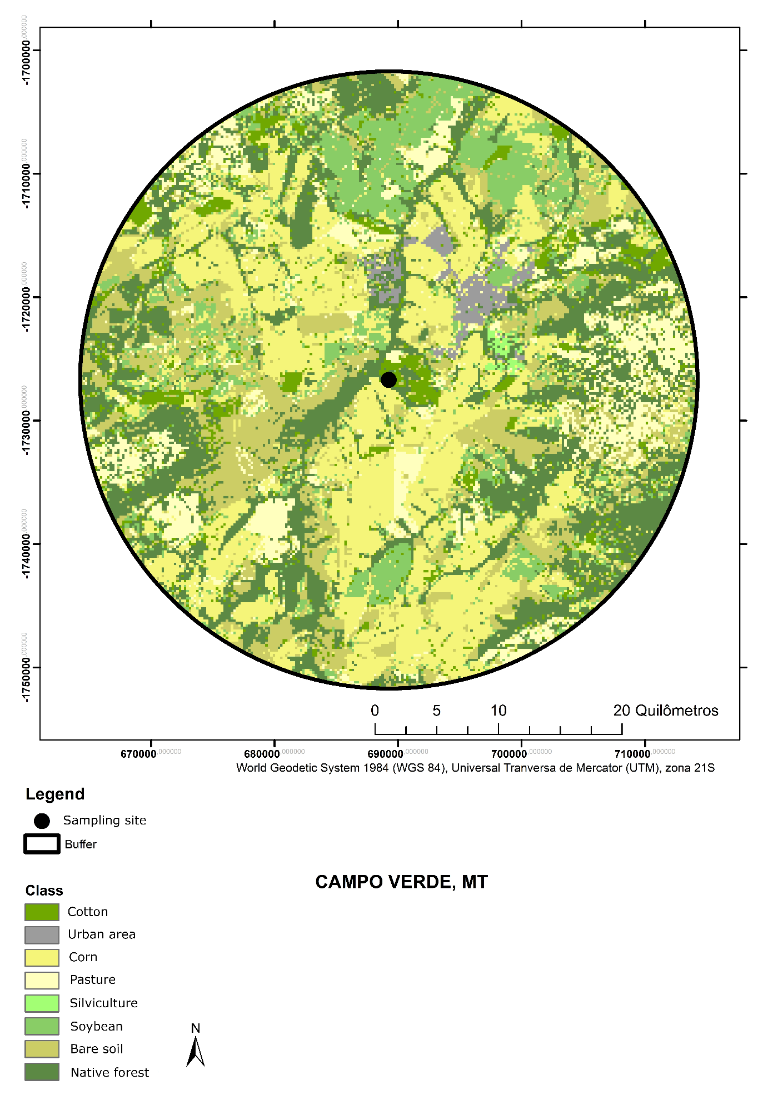


**Table S5F.** Landscape information by attributes present in a 25-km radius (1.96×10^9^ m^2^) in Campo Verde, Mato Grosso (MTCVC).

| Class | Land cover | Landscape Proportion | Edge length (m) | Edge density | Number of Patches | Mean patch area |
| --- | --- | --- | --- | --- | --- | --- |
| Soybean | 236480000 | 0.094592 | 2118400 | 0.001079033 | 1136 | 208169.0141 |
| Bare soil | 351680000 | 0.140672 | 2925600 | 0.00149019 | 1155 | 304484.8485 |
| Native forest | 463160000 | 0.185264 | 3220400 | 0.00164035 | 662 | 699637.4622 |
| Pasture | 206880000 | 0.082752 | 1730400 | 0.0008814 | 555 | 372756.7568 |
| Cotton | 127960000 | 0.051184 | 1645600 | 0.000838206 | 1259 | 101636.2192 |
| Water | 4240000 | 0.001696 | 38400 | 1.96E-05 | 7 | 605714.2857 |
| Urban area | 27720000 | 0.011088 | 174400 | 8.88E-05 | 18 | 1540000 |
| Corn | 545120000 | 0.218048 | 3018000 | 0.001537255 | 956 | 570209.205 |

**Figure S5G.** Land use and cover map obtained from a 25-km radius (1.96×10^9^ m^2^) of the sampled location in Campo Grande, Mato Grosso do Sul (MSCG).


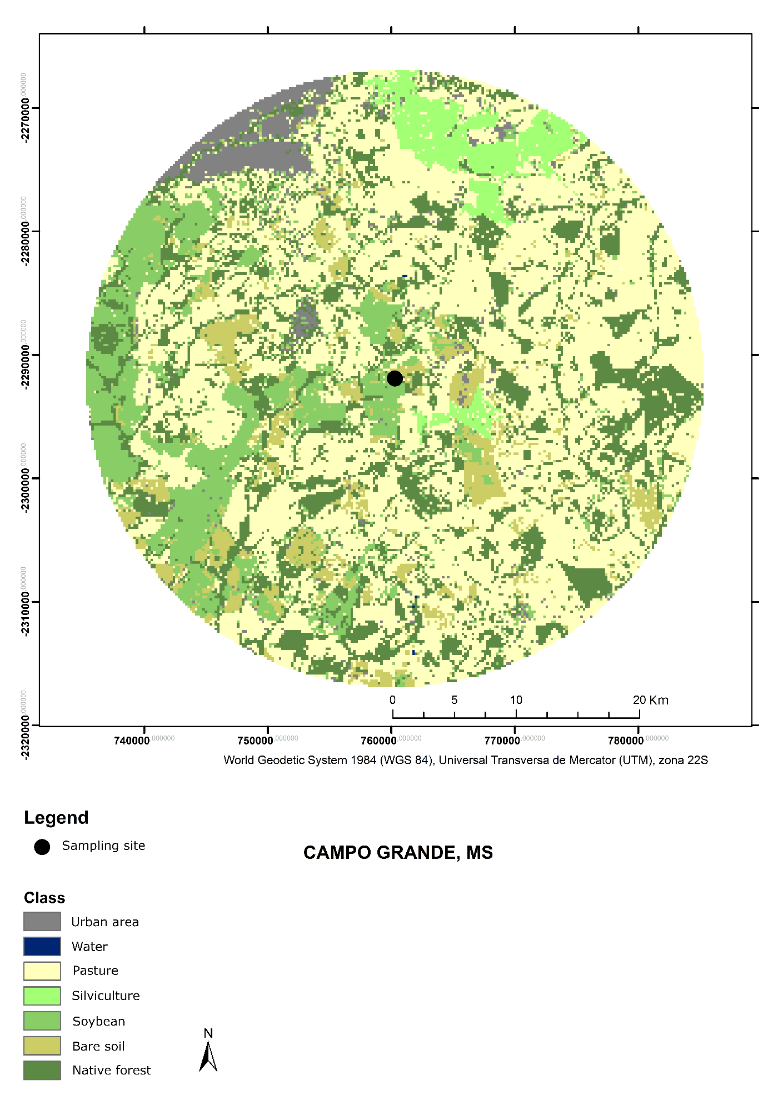


**Table S5G.** Landscape information by attributes present in 25 km radio (1.96×10^9^ m^2^) in Campo Grande, Mato Grosso do Sul (MSCG).

| Class | Land cover | Landscape Proportion | Edge length (m) | Edge density | Number of Patches | Mean patch area |
| --- | --- | --- | --- | --- | --- | --- |
| Soybean | 261920000 | 0.104768 | 1740400 | 0.000886458 | 868 | 301751.1521 |
| Bare soil | 137840000 | 0.055136 | 1092400 | 0.000556404 | 425 | 324329.4118 |
| Native forest | 401920000 | 0.160768 | 3396800 | 0.001730131 | 931 | 431707.841 |
| Pasture | 1006520000 | 0.402608 | 4464000 | 0.0022737 | 448 | 2246696.429 |
| Water | 240000 | 9.60E-05 | 4000 | 2.04E-06 | 4 | 60000 |
| Silviculture | 78000000 | 0.0312 | 242400 | 0.000123464 | 12 | 6500000 |
| Urban area | 76880000 | 0.030752 | 635600 | 0.000323737 | 429 | 179207.4592 |

**Figure S5H.** Land use and cover map obtained from a 25-km radius (1.96×10^9^ m^2^) of the sampled location in Mineiros, Goiás (GOMI).


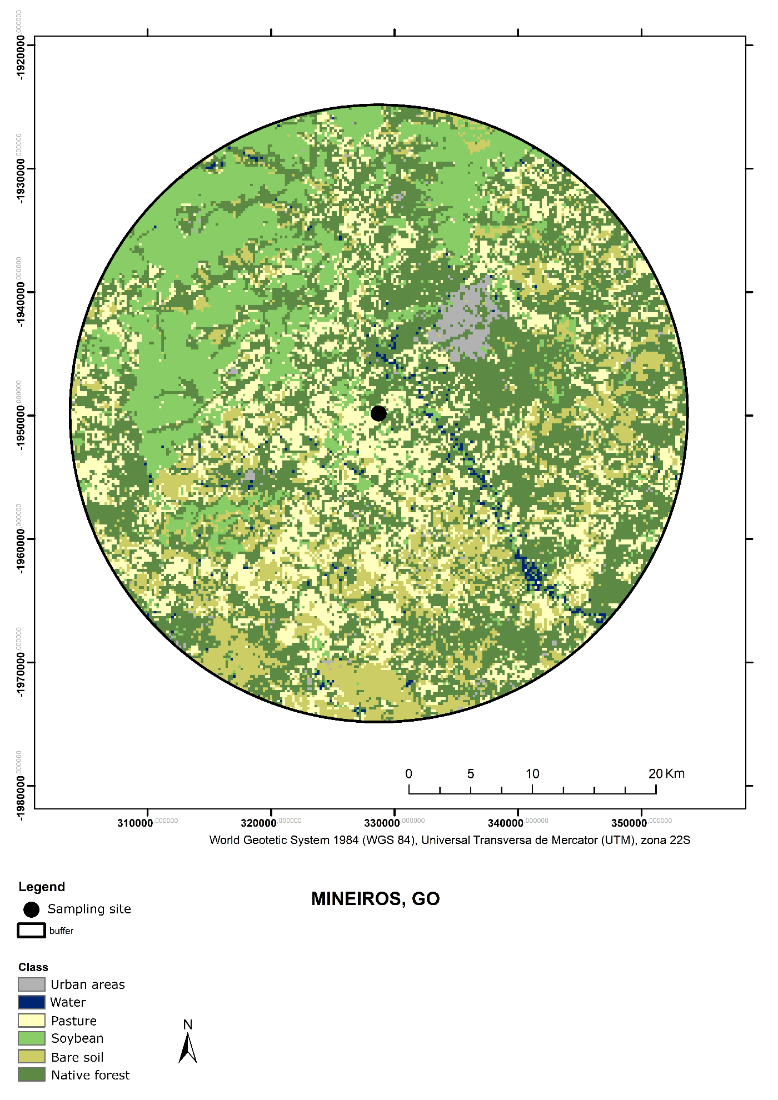


**Table S5H.** Landscape information by attributes present in 25 km radio (1.96×10^9^ m^2^) in Mineiros, Goiás (GOMI).

| Class | Land cover | Landscape Proportion | Edge length (m) | Edge density | Number of Patches | Mean patch area |
| --- | --- | --- | --- | --- | --- | --- |
| Soybean | 371600000 | 0.14864 | 1761600 | 0.000897439 | 560 | 663571.4286 |
| Bare soil | 289360000 | 0.115744 | 2582000 | 0.001315387 | 957 | 302361.5465 |
| Native forest | 798400000 | 0.31936 | 5404400 | 0.002753245 | 504 | 1584126.984 |
| Pasture | 459440000 | 0.183776 | 4267600 | 0.002174108 | 1114 | 412423.6984 |
| Water | 15040000 | 0.006016 | 240800 | 0.000122674 | 191 | 78743.4555 |
| Urban area | 29080000 | 0.011632 | 254800 | 0.000129807 | 159 | 182893.0818 |

**Figure S5I.** Land use and cover map obtained from a 25-km radius (1.96×10^9^ m^2^) of the sampled location in Araguarí, Minas Gerais (MGAR).


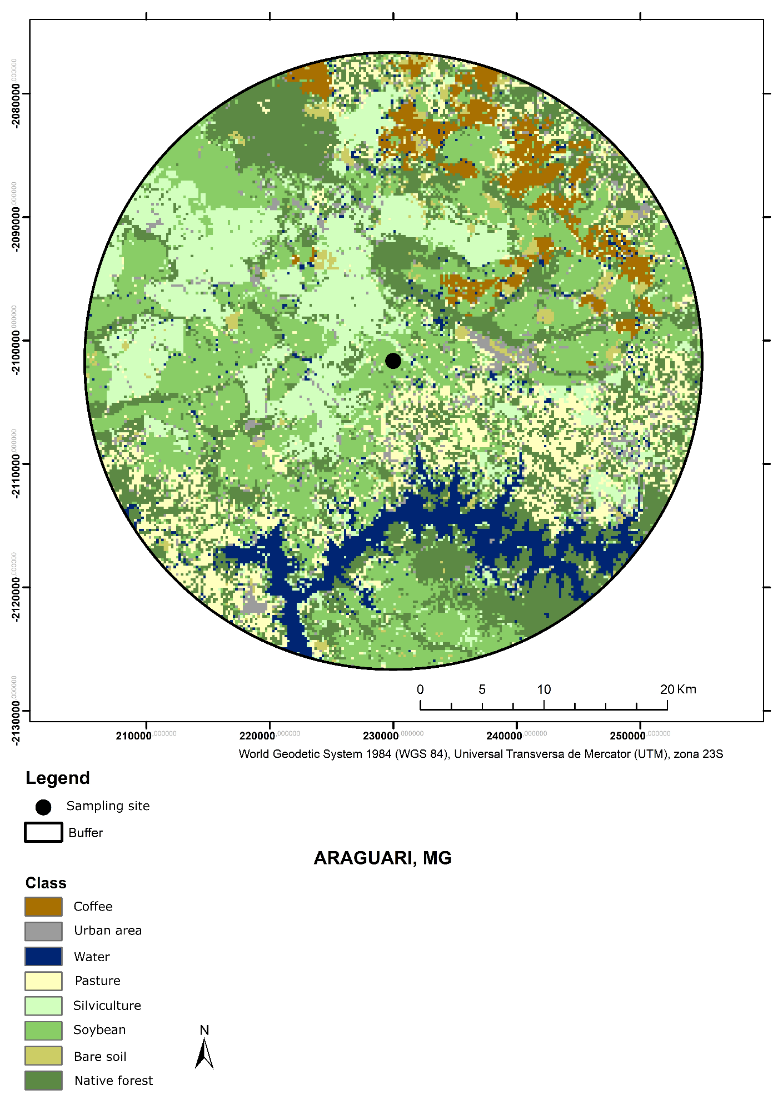


**Table S5I.** Landscape information by attributes present in 25 km radio (1.96×10^9^ m^2^) in Araguari Minas Gerais (MGAR).

| Class | Land cover | Landscape Proportion | Edge length (m) | Edge density | Number of Patches | Mean patch area |
| --- | --- | --- | --- | --- | --- | --- |
| Soybean | 547520000 | 0.219008 | 3186800 | 0.001623268 | 1001 | 546973.027 |
| Bare soil | 45360000 | 0.018144 | 486800 | 0.000247963 | 286 | 158601.3986 |
| Native forest | 487520000 | 0.195008 | 3766800 | 0.001918704 | 903 | 539889.258 |
| Pasture | 238360000 | 0.095344 | 2557600 | 0.001302771 | 896 | 266026.7857 |
| Water | 137240000 | 0.054896 | 963200 | 0.000490628 | 486 | 282386.8313 |
| Silviculture | 344400000 | 0.13776 | 2075200 | 0.00105705 | 861 | 400000 |
| Urban area | 72560000 | 0.029024 | 1069200 | 0.000544621 | 833 | 87106.84274 |
| Coffee | 90240000 | 0.036096 | 500800 | 0.000255094 | 38 | 2374736.842 |

**Figure S5J.** Land use and cover map obtained from a 25-km radius (1.96×10^9^ m^2^) of the sampled location in Casa Branca, São Paulo (SPCB).


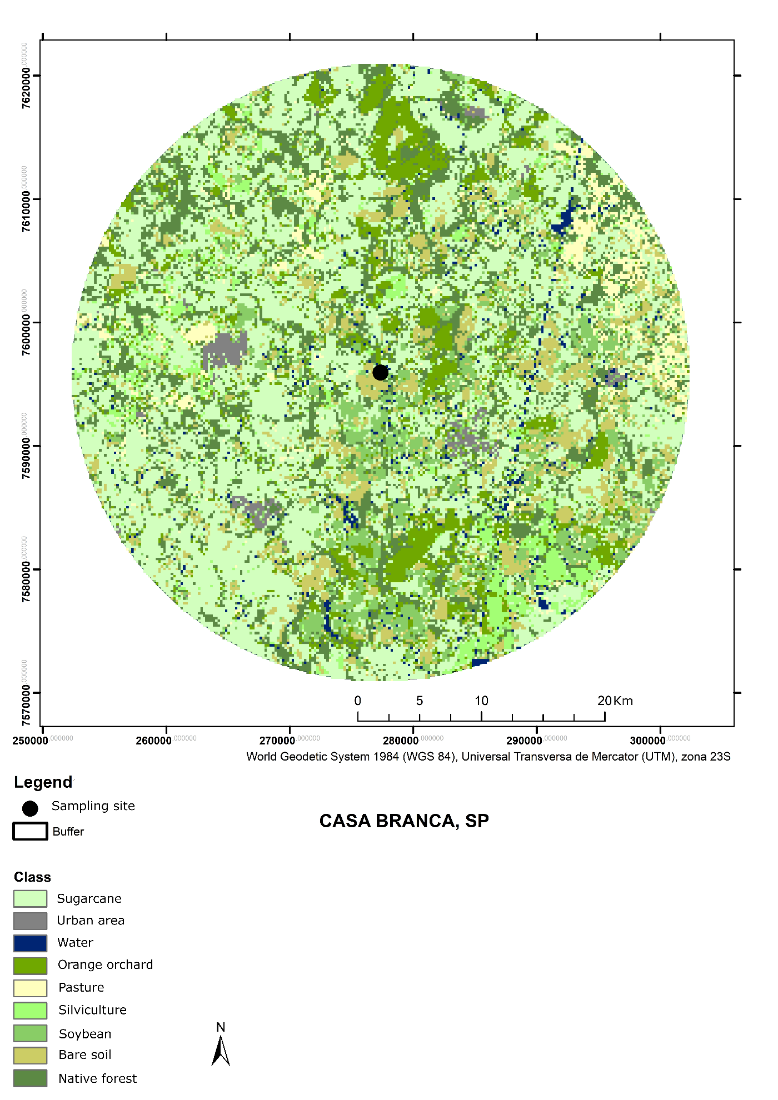


**Table S5J.** Landscape information by attributes present in 25 km radio (1.96×10^9^ m^2^) in Casa Branca, São Paulo (SPCB).

| Class | Land cover | Landscape Proportion | Edge length (m) | Edge density | Number of Patches | Mean patch area |
| --- | --- | --- | --- | --- | --- | --- |
| Soybean | 175200000 | 0.07008 | 2058400 | 0.00104783 | 1349 | 129873.9807 |
| Bare soil | 185760000 | 0.074304 | 1914400 | 0.000974527 | 930 | 199741.9355 |
| Native forest | 345920000 | 0.138368 | 3887600 | 0.001978986 | 1666 | 207635.054 |
| Pasture | 86360000 | 0.034544 | 1088800 | 0.000554255 | 691 | 124978.2923 |
| Orange orchard | 192480000 | 0.076992 | 2128000 | 0.00108326 | 1379 | 139579.4054 |
| Water | 32960000 | 0.013184 | 545200 | 0.000277535 | 516 | 63875.96899 |
| Sugarcane | 836880000 | 0.334752 | 5692400 | 0.002897721 | 923 | 906695.558 |
| Silviculture | 87880000 | 0.035152 | 1030000 | 0.000524322 | 729 | 120548.6968 |
| Urban area | 21000000 | 0.0084 | 169600 | 8.63E-05 | 39 | 538461.5385 |

**Figure S5K.** Land use and cover map obtained from a 25-km radius (1.96×10^9^ m^2^) of the sampled location in Pitanga, Paraná (PRPI).


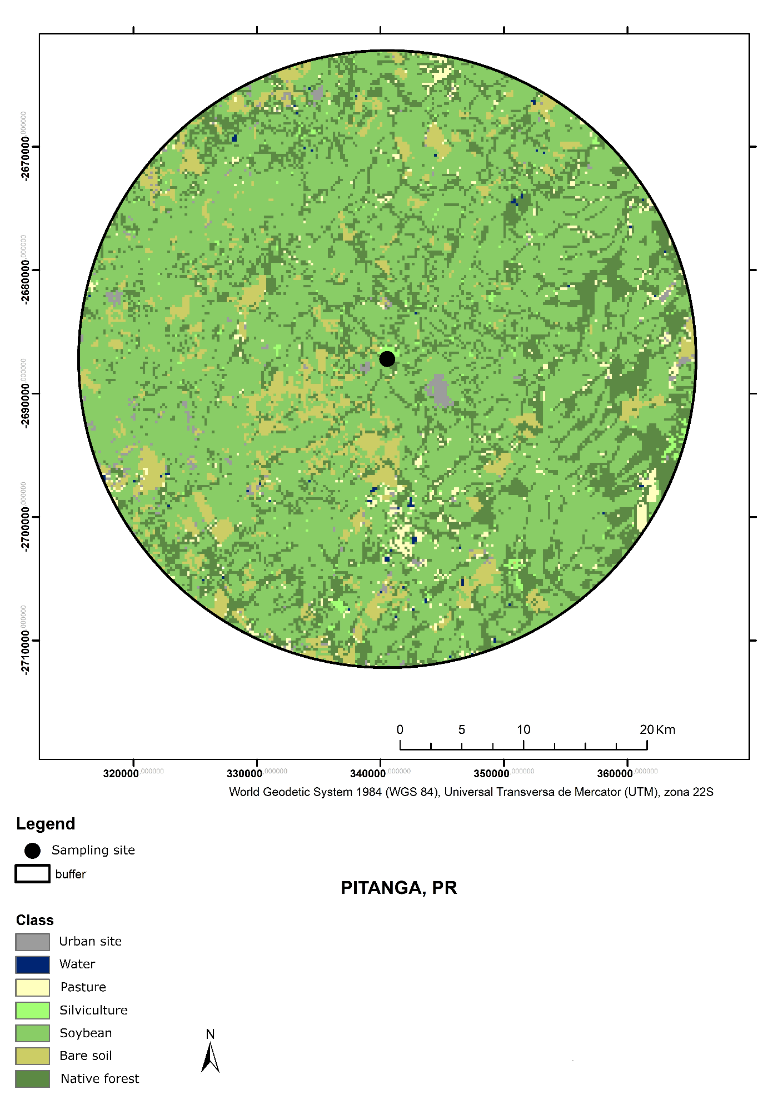


**Table S5L.** Landscape information by attributes present in 25 km radio (1.96×10^9^ m^2^) in Pitanga, Paraná (PRPI).

| Class | Land cover | Landscape Proportion | Edge length (m) | Edge density | Number of Patches | Mean patch area |
| --- | --- | --- | --- | --- | --- | --- |
| Soybean | 1300080000 | 0.520032 | 4768000 | 0.002428886 | 135 | 9630222.222 |
| Bare soil | 160560000 | 0.064224 | 1212000 | 0.00061741 | 333 | 482162.1622 |
| Native forest | 417200000 | 0.16688 | 4104400 | 0.002090839 | 1118 | 373166.3685 |
| Pasture | 44360000 | 0.017744 | 629200 | 0.000320523 | 449 | 98797.32739 |
| Water | 3600000 | 0.00144 | 57200 | 2.91E-05 | 55 | 65454.54545 |
| Silviculture | 15400000 | 0.00616 | 228400 | 0.00011635 | 208 | 74038.46154 |
| Urban area | 21840000 | 0.008736 | 280800 | 0.000143043 | 198 | 110303.0303 |

**Figure S5M.** Land use and cover map obtained from a 25-km radius (1.96×10^9^ m^2^) of the sampled location in Coxilha, RS (RSCO).


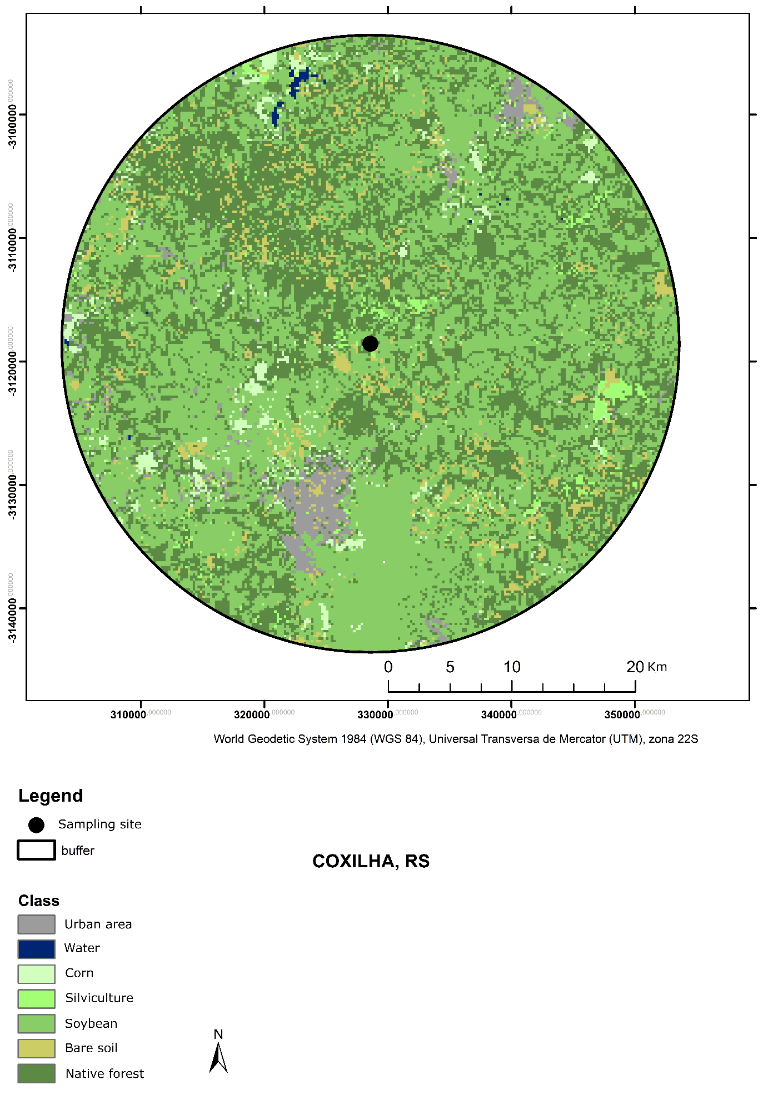


**Table S5M.** Landscape information by attributes present in 25 km radio (1.96×10^9^ m^2^) in Coxilha, RS (RSCO).

| Class | Land cover | Landscape Proportion | Edge length (m) | Edge density | Number of Patches | Mean patch area |
| --- | --- | --- | --- | --- | --- | --- |
| Soybean | 1046880000 | 0.418752 | 5574800 | 0.002839881 | 385 | 2719168.831 |
| Bare soil | 101840000 | 0.040736 | 1342800 | 0.000684041 | 845 | 120520.7101 |
| Native forest | 670160000 | 0.268064 | 5754000 | 0.002931168 | 975 | 687343.5897 |
| Water | 3240000 | 0.001296 | 30800 | 1.57E-05 | 13 | 249230.7692 |
| Silviculture | 31480000 | 0.012592 | 432800 | 0.000220474 | 344 | 91511.62791 |
| Urban area | 59080000 | 0.023632 | 556800 | 0.000283642 | 283 | 208763.2509 |
| Corn | 50360000 | 0.020144 | 626000 | 0.000318893 | 424 | 8773.5849 |

**Appendix S6.** Percentage of variance explained in the Principal Component Analysis (PCA) of 12 agricultural landscapes in Brazil (see appendix S1 and S5) for (A) 15 landscape variables (i.e. soybean, forest, and silviculture area) and (B) 19 environmental variables (e.g., annual mean temperature, mean diurnal temperature range, and isothermality).


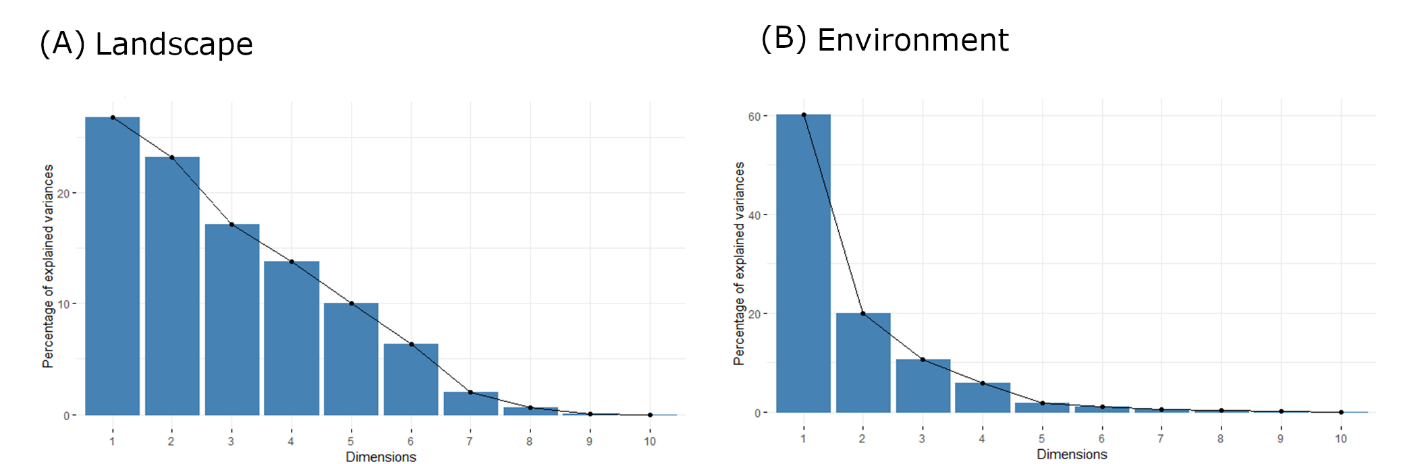


**Table S6.** Site bioclimatic information according to WorldClim variables at <http://www.worldclim.org/bioclim>

|  |  | WorldClim variables (WC) | | | | | | | | | | | | | | | | | | |
| --- | --- | --- | --- | --- | --- | --- | --- | --- | --- | --- | --- | --- | --- | --- | --- | --- | --- | --- | --- | --- |
| POP | Elevation (m) | 01 | 02 | 03 | 04 | 05 | 06 | 07 | 08 | 09 | 10 | 11 | 12 | 13 | 14 | 15 | 16 | 17 | 18 | 19 |
| CERUB | 63 | 272 | 100 | 80 | 606 | 335 | 211 | 124 | 269 | 277 | 280 | 264 | 805 | 213 | 1 | 108 | 529 | 5 | 84 | 189 |
| PITEB | 97 | 274 | 114 | 70 | 1015 | 362 | 200 | 162 | 266 | 278 | 290 | 265 | 1374 | 316 | 6 | 92 | 805 | 31 | 97 | 154 |
| BACO | 704 | 236 | 137 | 72 | 1063 | 326 | 137 | 189 | 241 | 221 | 248 | 221 | 845 | 155 | 1 | 85 | 444 | 5 | 216 | 5 |
| MTCV | 646 | 225 | 130 | 68 | 1479 | 309 | 118 | 191 | 235 | 202 | 235 | 201 | 1757 | 296 | 7 | 73 | 820 | 35 | 809 | 78 |
| MSCG | 496 | 234 | 113 | 69 | 1672 | 307 | 144 | 163 | 248 | 213 | 249 | 209 | 1434 | 223 | 35 | 50 | 590 | 119 | 570 | 184 |
| GOMI | 740 | 230 | 125 | 70 | 1306 | 309 | 132 | 177 | 238 | 214 | 242 | 209 | 1679 | 309 | 17 | 71 | 792 | 59 | 440 | 91 |
| MGAR | 976 | 206 | 113 | 68 | 1644 | 275 | 111 | 164 | 218 | 184 | 220 | 180 | 1478 | 277 | 9 | 80 | 770 | 30 | 654 | 50 |
| SPCB | 675 | 206 | 121 | 64 | 2209 | 284 | 97 | 187 | 228 | 174 | 228 | 174 | 1297 | 235 | 17 | 72 | 646 | 71 | 646 | 83 |
| PRPI | 660 | 197 | 119 | 61 | 2722 | 288 | 96 | 192 | 229 | 172 | 229 | 160 | 1618 | 200 | 83 | 27 | 535 | 297 | 535 | 361 |
| RSCO | 561 | 186 | 106 | 54 | 3071 | 290 | 94 | 196 | 201 | 198 | 220 | 146 | 1725 | 182 | 119 | 12 | 501 | 381 | 437 | 405 |

All calculations were made in an R script and rounded for presentation

**Appendix S7**. Clustering analyses based on SNP markers and geographic distribution across 10 collection sites. Along the *x*-axis of each plot, each vertical bar represents a specimen; along the *y*-axis, the membership coefficient of a specimen. The best value for K (K= 4) was calculated according to the ΔK method (Evanno et al., 2005). Structure analysis reveals a pattern of admixture.


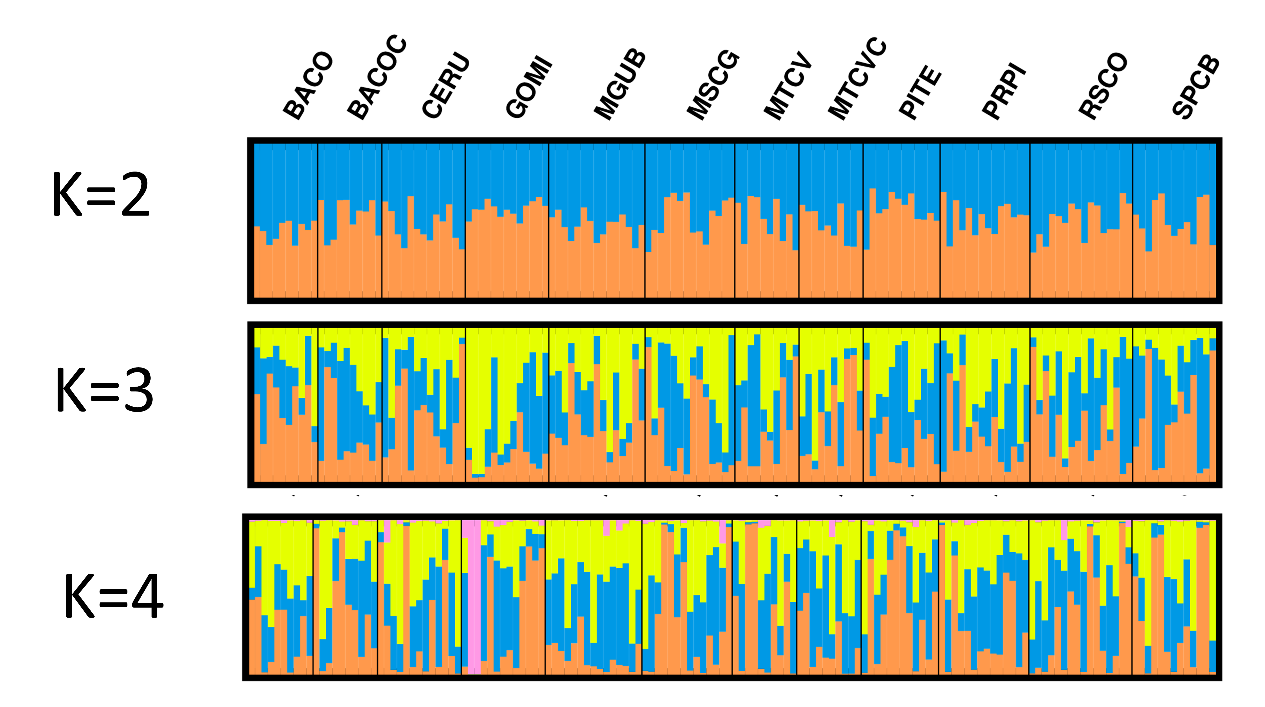


Reference:

Evanno, G., Regnaut, S., & Goudet, J. (2005). Detecting the number of clusters of individuals using the software STRUCTURE: a simulation study. Molecular Ecology, 14(8), 2611-2620.
